# Foundation Model‐Enabled Pathology‐Informed MRI Model for Preoperative Prediction of Vessels Encapsulating Tumor Clusters in Resectable Hepatocellular Carcinoma: A Multicenter Retrospective Study

**DOI:** 10.1002/advs.78135

**Published:** 2026-10-18

**Authors:** Zhehan Shen, Wanmin Liu, Xiaoming Li, Wenjie Zou, Yiman Li, Jie Yuan, Yasen Yimit, Shihang Wang, Linhao Qu, Hui Dong, Wei Chen, Ningyang Jia, Peijun Wang

**Affiliations:** ^1^ Department of Radiology Ruijin Hospital College of Health Science and Technology Shanghai Jiao Tong University School of Medicine Shanghai China; ^2^ Department of Radiology Ruijin Hospital Shanghai Jiao Tong University School of Medicine Shanghai China; ^3^ 7T Magnetic Resonance Translational Medicine Research Center Department of Radiology The First Affiliated Hospital of Army Medical University Chongqing China; ^4^ Department of Radiology School of Medicine Tongji Hospital Tongji University Shanghai China; ^5^ Department of Radiology Shuguang Hospital Affiliated to Shanghai University of Traditional Chinese Medicine Shanghai China; ^6^ Department of Radiology The First People's Hospital of Kashi Prefecture Kashi Xinjiang China; ^7^ Faculty of Applied Sciences Macao Polytechnic University Macao China; ^8^ Department of Biomedical Informatics Harvard Medical School Boston Massachusetts USA; ^9^ Department of Pathology Eastern Hepatobiliary Surgery Hospital The Third Affiliated Hospital of Naval Medical University Shanghai China; ^10^ Department of Radiology Eastern Hepatobiliary Surgery Hospital The Third Affiliated Hospital of Naval Medical University Shanghai China

**Keywords:** digital pathology, foundation model, hepatocellular carcinoma, magnetic resonance imaging, multimodal learning, vessels encapsulating tumor clusters

## Abstract

Vessels encapsulating tumor clusters (VETC) are an aggressive vascular phenotype of hepatocellular carcinoma that is usually determined after surgery. A foundation model‐enabled framework for pathology‐informed VETC prediction on pretreatment magnetic resonance imaging (MRI), termed PIVOT, uses paired preoperative multiparametric MRI, postoperative hematoxylin and eosin whole‐slide images, and CD34 immunohistochemistry during model development, while inference requires only preoperative MRI. PIVOT‐MRI uses morphologic and vascular embedding alignment, and PIVOT‐Clinical combines the PIVOT‐MRI score with tumor size and alpha‐fetoprotein. The multicenter retrospective study includes 2052 patients in a training cohort, an internal test cohort, and four external validation cohorts. PIVOT‐MRI achieves an area under the receiver operating characteristic curve of 0.887 in the internal test cohort and 0.839‐0.861 across external cohorts. PIVOT‐Clinical increases the internal‐test area under the curve to 0.904 and PIVOT‐Clinical‐defined groups are associated with different two‐year recurrence risks. Removing both histopathologic alignment branches results in the largest ablation‐related performance decrease. Exploratory radiotranscriptomic analyses showed that higher PIVOT‐MRI scores were associated with angiogenesis‐related, endothelial, and proliferative transcriptional programs. PIVOT enables noninvasive preoperative assessment of pathology‐defined vascular aggressiveness in resectable hepatocellular carcinoma while preserving an MRI‐only clinical inference pathway.

## Graphical abstract

PIVOT uses pathology‐derived morphologic and vascular representations from postoperative H&E and CD34 whole‐slide images to guide model development, while clinical inference requires only preoperative multiparametric MRI. Across five centers, PIVOT enabled accurate preoperative assessment of vessels encapsulating tumor clusters, stratified patients by early‐recurrence risk, and showed exploratory radiotranscriptomic associations with angiogenesis‐related, endothelial, and proliferative transcriptional programs.

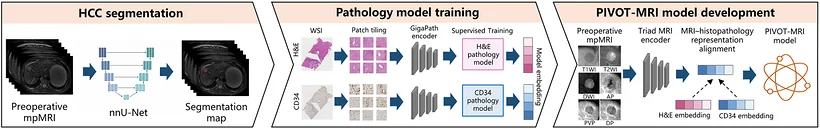

## 1 Introduction

Hepatocellular carcinoma (HCC) remains a major global health burden and is among the leading causes of cancer‐related mortality worldwide [1]. Current treatment allocation is largely guided by tumor burden, liver function, performance status, and stage‐based systems such as the Barcelona Clinic Liver Cancer (BCLC) strategy and contemporary practice guidelines [2, 3]. Although surveillance and advances in locoregional, surgical, and systemic therapies have improved the management of selected patients, recurrence after curative‐intent resection, ablation, or transplantation remains common. This clinical heterogeneity is only partly explained by conventional staging variables and reflects the marked histologic, vascular, molecular, and biological diversity of HCC [4, 5]. Identifying aggressive tumor phenotypes during the pretherapeutic work‐up could therefore refine recurrence‐risk estimation and define biologically informed subgroups for treatment evaluation beyond stage‐based allocation.

Vessels encapsulating tumor clusters (VETC) represent a distinctive vascular pattern in HCC, in which sinusoid‐like vessels surround and encapsulate tumor cell clusters [6, 7]. Unlike conventional microvascular invasion, VETC has been linked to a nonclassical route of hematogenous dissemination and tumor spread [6]. Biologically, VETC reflects an angiogenic and endothelial‐remodeling program that forms vascular niches around tumor nests and may facilitate collective hematogenous dissemination [6, 8, 9]. In a large multi‐institutional cohort of resected HCCs, VETC was significantly associated with several clinical and pathological features [7]. VETC was also associated with early recurrence, disease‐free survival, and overall survival at multivariable analysis [7, 8]. These findings support VETC as a clinically meaningful marker of aggressive vascular biology in HCC and as a potential basis for risk stratification beyond conventional staging variables.

Pretherapeutic characterization of VETC, an aggressive histopathologic phenotype associated with early recurrence and poor survival, could enable recurrence‐risk stratification before surgery and identify a biologically defined subgroup for treatment‐stratification studies. Conventional computed tomography (CT) and magnetic resonance imaging (MRI) studies have explored VETC‐related imaging features, including tumor size, intratumoral necrosis, intratumoral arteries, capsule appearance, arterial enhancement patterns, and tumor margin or peritumoral parenchymal abnormalities [10, 11, 12, 13]. These radiologic imaging features are clinically intuitive, but their qualitative assessment is reader dependent and limited in repeatability; moreover, visual feature assessment may be insufficient to capture the high‐dimensional intensity, texture, and enhancement patterns associated with VETC [14, 15, 16, 17]. Radiomics and deep learning studies have extended this approach by extracting high‐dimensional imaging features for VETC prediction and prognosis assessment [14, 15, 16, 17]. In most previous studies, VETC prediction has been approached by extracting radiomics or deep learning features from pretreatment imaging and optimizing these models against the pathologic VETC label [14, 15, 16, 17]. Yu et al. further developed deep learning radiopathomics models that combined contrast‐enhanced MRI with representativehematoxylin and eosin (H&E)‐stained pathologic image patches, suggesting that selected microscopic tissue morphology may add information beyond imaging‐derived phenotypes [14]. However, because such models require pathologic images at prediction, their application is inherently limited to settings in which surgical specimens are already available and therefore does not meet the clinical need for noninvasive preoperative MRI‐based VETC assessment.

This gap highlights the need for a model development strategy that can use postoperative histology without requiring pathologic images at preoperative assessment. Paired pretreatment MRI, postoperative H&E whole‐slide images, and CD34 immunohistochemistry can provide complementary macroscopic, morphologic, and vascular information from the same tumor. MRI depicts lesion morphology and enhancement‐related phenotypes, H&E pathology captures histologic tumor architecture, cellular morphology, and the tumor‐stromal microenvironment, and CD34 immunohistochemistry directly highlights the vascular organization used to define VETC [18, 19, 20, 21, 22]. Because H&E and CD34 pathology are not available for pretreatment decision‐making, they can guide MRI model development rather than serving as required inputs in clinical use. This strategy also poses a representational challenge, because multiparametric MRI and high‐resolution pathology images differ substantially in dimensionality, spatial scale, staining biology, and data structure. Foundation models pretrained on large‐scale medical images can learn transferable, domain‐specific representations and provide efficient feature extraction for heterogeneous radiologic and pathologic data [21, 23, 24]. Qu et al. developed a foundation model‐enabled multimodal framework that integrated pathology, radiology, and clinical information for prognostic prediction in patients with colorectal cancer with incomplete modalities [21]. Li et al. reported a multimodal deep learning system in which radiologic and pathologic representations were jointly leveraged for noninvasive breast cancer diagnosis [24]. Saueressig et al. fused pathology and radiology foundation models for WHO 2021 glioma subtyping, integrating whole‐slide histology, and multiparametric MRI representations in a multimodal classification framework [23]. These studies support the feasibility of using foundation‐model representations to process heterogeneous medical images and to incorporate histopathologic information during model development, while preserving VETC assessment from pretreatment MRI in clinical use.

Building on this rationale, in this multicenter study, we developed PIVOT (Pathology‐Informed VETC prediction On preTreatment MRI), a foundation model‐enabled, pathology‐informed MRI framework for preoperative MRI‐based assessment of VETC status in HCC. The central objective was to enable VETC risk prediction from preoperative MRI alone, while using paired postoperative H&E whole‐slide pathology and CD34 immunohistochemistry to incorporate morphologic and vascular histopathologic information during model development. The MRI model was termed PIVOT‐MRI, and the final clinical model, termed PIVOT‐Clinical, incorporated PIVOT‐MRI score, and routinely available preoperative clinical variables. The performance of PIVOT‐MRI and PIVOT‐Clinical was evaluated across internal and external validation cohorts using diagnostic discrimination, calibration, and decision‐curve analysis. PIVOT‐Clinical was further evaluated for 2‐year recurrence‐risk stratification by applying the VETC classification cutoff unchanged across the validation cohorts. Furthermore, PIVOT‐MRI was compared with MRI‐based deep learning comparator models, GigaPath‐H&E, and GigaPath‐CD34 across validation cohorts. To enhance interpretability, SHAP analysis was used to assess predictor contributions in PIVOT‐Clinical, model‐attended MRI regions were visualized using activation maps, and PIVOT‐MRI‐derived risk scores were examined in relation to exploratory transcriptomic programs and immune‐cell abundance. We aimed to develop a clinically applicable approach in which pathology‐informed alignment guides model development and PIVOT‐Clinical provides a patient‐level probability of VETC before surgery.

## 2 Results

### 2.1 Baseline Characteristics

The overall study design, model‐development strategy, clinical deployment pathway, and validation framework are summarized in Figure 1. Detailed baseline characteristics are presented in Main Table 1, and the full names of the participating hospitals are provided in Methods S1.

**FIGURE 1:**
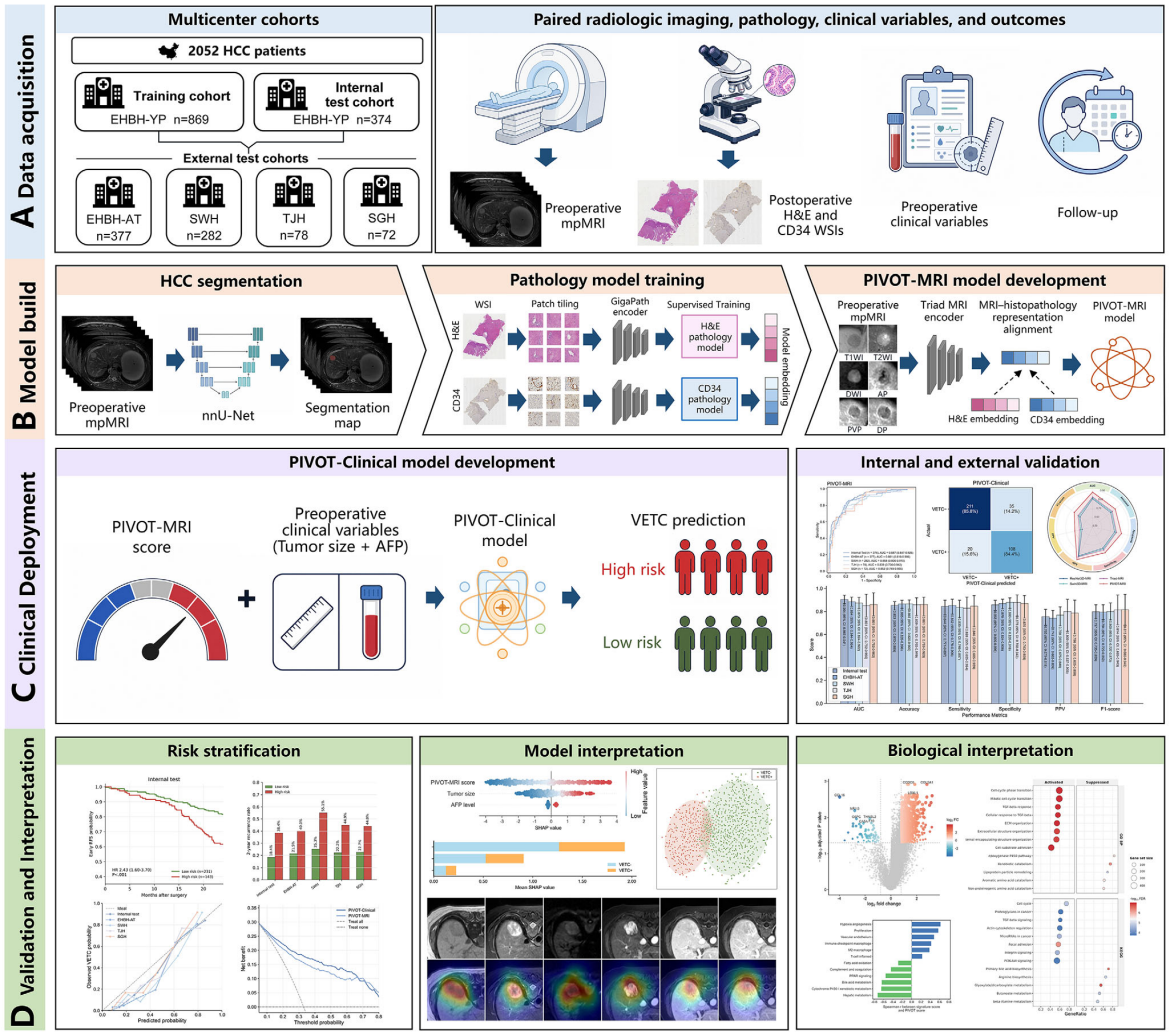
Multicenter study design and PIVOT framework for preoperative VETC assessment. (A) Five‐center cohort acquisition and paired preoperative multiparametric MRI, postoperative H&E and CD34 whole‐slide images, preoperative clinical variables, and follow‐up. (B) Model development comprised automated HCC segmentation, stain‐specific pathology model training, and MRI‐histopathology representation alignment. Six MRI sequences including T1‐weighted imaging, T2‐weighted imaging, diffusion‐weighted imaging, arterial phase, portal venous phase, and delayed phase were used. (C) The PIVOT‐MRI score was combined with tumor size and AFP to generate the PIVOT‐Clinical probability estimate and risk groups. (D) Internal and four‐center external validation included diagnostic performance, calibration, decision‐curve analysis, recurrence association, model interpretation, and exploratory radiotranscriptomic analyses. AFP, alpha‐fetoprotein; AP, arterial phase; DP, delayed phase; DWI, diffusion‐weighted imaging; HCC, hepatocellular carcinoma; H&E, hematoxylin and eosin; mpMRI, multiparametric MRI; PVP, portal venous phase; VETC, vessels that encapsulate tumor clusters; WSI, whole‐slide image.

**TABLE 1:** Patient Characteristics according to Cohort and VETC Status.

Characteristic	Training cohort (EHBH‐YP; n = 869)	VETC‐positive in training (n = 297)	VETC‐negative in training (n = 572)	*p* value*	Internal test (EHBH‐YP; n = 374)	EHBH‐AT (n = 377)	SWH (n = 282)	TJH (n = 78)	SGH (n = 72)
VETC‐positive, n (%)	297 (34.2)	297 (100.0)	0 (0.0)		128 (34.2)	115 (30.5)	91 (32.3)	29 (37.2)	26 (36.1)
VETC‐negative, n (%)	572 (65.8)	0 (0.0)	572 (100.0)		246 (65.8)	262 (69.5)	191 (67.7)	49 (62.8)	46 (63.9)
Age, years	56.2 ± 10.0	55.8 ± 10.8	56.4 ± 9.6	0.420	55.4 ± 11.3	56.2 ± 10.9	53.9 ± 11.0	61.6 ± 10.7	57.8 ± 10.6
Sex, female	126 (14.5)	41 (13.8)	85 (14.9)	0.675	60 (16.0)	70 (18.6)	54 (19.1)	14 (17.9)	13 (18.1)
Sex, male	743 (85.5)	256 (86.2)	487 (85.1)		314 (84.0)	307 (81.4)	228 (80.9)	64 (82.1)	59 (81.9)
Maximum tumor diameter, cm	3.0 (2.1–4.6)	3.4 (2.3–5.0)	2.7 (2.0–4.2)	<0.001	3.0 (2.1–4.5)	3.5 (2.3–5.8)	4.0 (3.0–7.0)	4.4 (3.0–8.0)	3.7 (2.4–6.0)
Tumor number, 1	783 (90.1)	266 (89.6)	517 (90.4)	0.700	328 (87.7)	331 (87.8)	257 (91.1)	70 (89.7)	64 (88.9)
Tumor number, ≥2	86 (9.9)	31 (10.4)	55 (9.6)		46 (12.3)	46 (12.2)	25 (8.9)	8 (10.3)	8 (11.1)
HBV infection, present	702 (80.8)	242 (81.5)	460 (80.4)	0.706	297 (79.4)	275 (72.9)	246 (87.2)	48 (61.5)	54 (75.0)
HBV infection, absent	167 (19.2)	55 (18.5)	112 (19.6)		77 (20.6)	102 (27.1)	36 (12.8)	30 (38.5)	18 (25.0)
Liver cirrhosis, present	572 (65.8)	203 (68.4)	369 (64.5)	0.258	253 (67.6)	218 (57.8)	154 (54.6)	61 (78.2)	45 (62.5)
Liver cirrhosis, absent	297 (34.2)	94 (31.6)	203 (35.5)		121 (32.4)	159 (42.2)	128 (45.4)	17 (21.8)	27 (37.5)
ALT level, ≤50 U/L	733 (84.3)	253 (85.2)	480 (83.9)	0.625	310 (82.9)	270 (71.6)	228 (80.9)	59 (75.6)	55 (76.4)
ALT level, >50 U/L	136 (15.7)	44 (14.8)	92 (16.1)		64 (17.1)	107 (28.4)	54 (19.1)	19 (24.4)	17 (23.6)
AST level, ≤40 U/L	713 (82.0)	244 (82.2)	469 (82.0)	0.953	282 (75.4)	238 (63.1)	182 (64.5)	47 (60.3)	49 (68.1)
AST level, >40 U/L	156 (18.0)	53 (17.8)	103 (18.0)		92 (24.6)	139 (36.9)	100 (35.5)	31 (39.7)	23 (31.9)
AFP level, ≤100 ng/mL	572 (65.8)	179 (60.3)	393 (68.7)	0.013	205 (54.8)	204 (54.1)	133 (47.2)	55 (70.5)	43 (59.7)
AFP level, >100 ng/mL	297 (34.2)	118 (39.7)	179 (31.3)		169 (45.2)	173 (45.9)	149 (52.8)	23 (29.5)	29 (40.3)

The study cohort included 2052 patients with HCC across a training cohort, an internal test cohort, and four external validation cohorts. The training cohort included 869 patients from EHBH‐YP, of whom 297 (34.2%) were VETC‐positive and 572 (65.8%) were VETC‐negative. The internal test cohort included 374 patients from EHBH‐YP, with 128 (34.2%) VETC‐positive tumors. The external validation cohorts included EHBH‐AT (n = 377; VETC‐positive, 115 [30.5%]), SWH (n = 282; VETC‐positive, 91 [32.3%]), TJH (n = 78; VETC‐positive, 29 [37.2%]), and SGH (n = 72; VETC‐positive, 26 [36.1%]). Patient selection and cohort allocation are shown in Figure 2.

**FIGURE 2:**
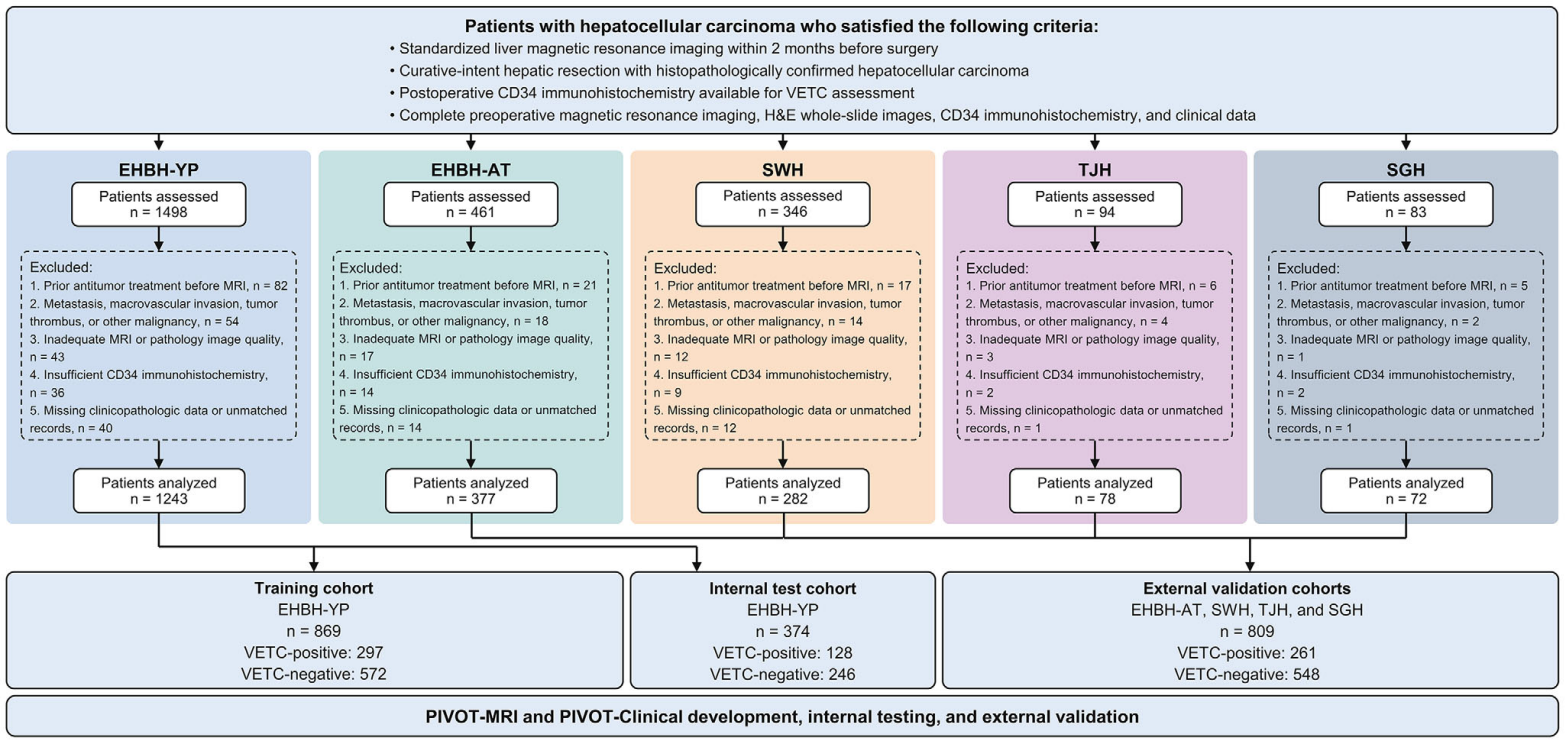
Patient selection and cohort allocation. Patients with hepatocellular carcinoma who fulfilled the prespecified imaging, surgical, pathologic, and data‐completeness criteria were assessed at five centers. The EHBH‐YP cohort was divided at the patient level into a training cohort (n = 869) and an internal test cohort (n = 374). EHBH‐AT (n = 377), SWH (n = 282), TJH (n = 78), and SGH (n = 72) formed four independent external validation cohorts, yielding 2052 analyzed patients. EHBH‐AT, Eastern Hepatobiliary Surgery Hospital‐Anting; EHBH‐YP, Eastern Hepatobiliary Surgery Hospital‐Yangpu; SGH, Shuguang Hospital; SWH, Southwest Hospital; TJH, Tongji Hospital; VETC, vessels that encapsulate tumor clusters.

Because the EHBH‐YP cohort was randomly divided at the patient level, VETC prevalence was balanced between the training and internal test cohorts. External validation cohorts were kept unchanged to evaluate between‐center generalizability across different clinical settings.

In the training cohort, VETC‐positive tumors were larger than VETC‐negative tumors, with a median maximum diameter of 3.4 vs. 2.7 cm (P < 0.001). Alpha‐fetoprotein (AFP) level greater than 100 ng/mL was more frequent in VETC‐positive tumors (p = 0.013), whereas age, sex, tumor number, HBV infection, liver cirrhosis, ALT level, and AST level did not differ significantly between VETC‐positive and VETC‐negative patients. In logistic regression analysis, maximum tumor diameter was associated with VETC positivity in both univariable analysis (OR, 1.19; 95% CI, 1.11–1.28; P < 0.001) and multivariable analysis (OR, 1.14; 95% CI, 1.03–1.27; p = 0.009). AFP level greater than 100 ng/mL was also associated with VETC positivity in univariable analysis (OR, 1.45; 95% CI, 1.08–1.94; p = 0.013) and remained significant in multivariable analysis (OR, 1.61; 95% CI, 1.07–2.41; p = 0.022). The PIVOT‐MRI score was independently associated with VETC positivity, with a multivariable OR of 2.30 per 0.1 increase (95% CI, 2.07–2.56; P < 0.001). Detailed results of the univariable and multivariable logistic regression analyses are presented in Main Table 2.

**TABLE 2:** Logistic Regression Analysis for VETC Prediction.

Variable	Univariable OR	*p* value	Multivariable OR	*p* value
Age, continuous	0.99 (0.98, 1.01)	0.403		
Sex, female vs.male	0.92 (0.61, 1.37)	0.675		
Maximum tumor diameter, continuous	1.19 (1.11, 1.28)	<0.001	1.14 (1.03, 1.27)	0.009
Tumor number, ≥2 vs.1	1.10 (0.69, 1.74)	0.700		
HBV infection, absent vs.present	0.93 (0.65, 1.34)	0.706		
Liver cirrhosis, absent vs.present	0.84 (0.62, 1.13)	0.258		
ALT level, >50 vs.≤50 U/L	0.91 (0.61, 1.34)	0.625		
AST level, >40 vs.≤40 U/L	0.99 (0.69, 1.43)	0.953		
AFP level, >100 vs.≤100 ng/mL	1.45 (1.08, 1.94)	0.013	1.61 (1.07, 2.41)	0.022
PIVOT‐MRI score, per 0.1	2.31 (2.08, 2.57)	<0.001	2.30 (2.07, 2.56)	<0.001

### 2.2 Performance of PIVOT‐MRI for Preoperative VETC Prediction

The automated 3D nnU‐Net pipeline localized the predefined index tumor in 364 of 374 patients in the internal test cohort (97.3%; 95% CI, 95.1‐98.5) and in 362 of 377 (96.0%), 275 of 282 (97.5%), 76 of 78 (97.4%), and 69 of 72 (95.8%) patients in the EHBH‐AT, SWH, TJH, and SGH cohorts. Manual fallback was required for the remaining 10 (2.7%), 15 (4.0%), 7 (2.5%), 2 (2.6%), and 3 (4.2%) patients, all of whom were retained in the final performance analyses. Liver segmentation was assessed in all patients and tumor segmentation in successfully localized cases. Mean liver and tumor Dice similarity coefficients (DSC) were 0.957 and 0.873 in the internal test cohort and ranged from 0.932 to 0.961 and 0.845 to 0.876 across the external cohorts (Table S2).

PIVOT‐MRI achieved an AUC of 0.887 (95% CI, 0.847–0.926) in the internal test cohort, with an accuracy of 0.821 (95% CI, 0.779–0.856), sensitivity of 0.828 (95% CI, 0.753–0.884), and specificity of 0.817 (95% CI, 0.764–0.860). External‐cohort AUCs were 0.861 (95% CI, 0.816–0.906) for EHBH‐AT, 0.858 (95% CI, 0.806–0.910) for SWH, 0.839 (95% CI, 0.736–0.942) for TJH, and 0.852 (95% CI, 0.749–0.955) for SGH, with sensitivities of 0.793–0.826 and specificities of 0.837–0.853. Multicenter performance and ablation results are summarized in Figure 3, with complete operating characteristics provided in Table S3.

**FIGURE 3:**
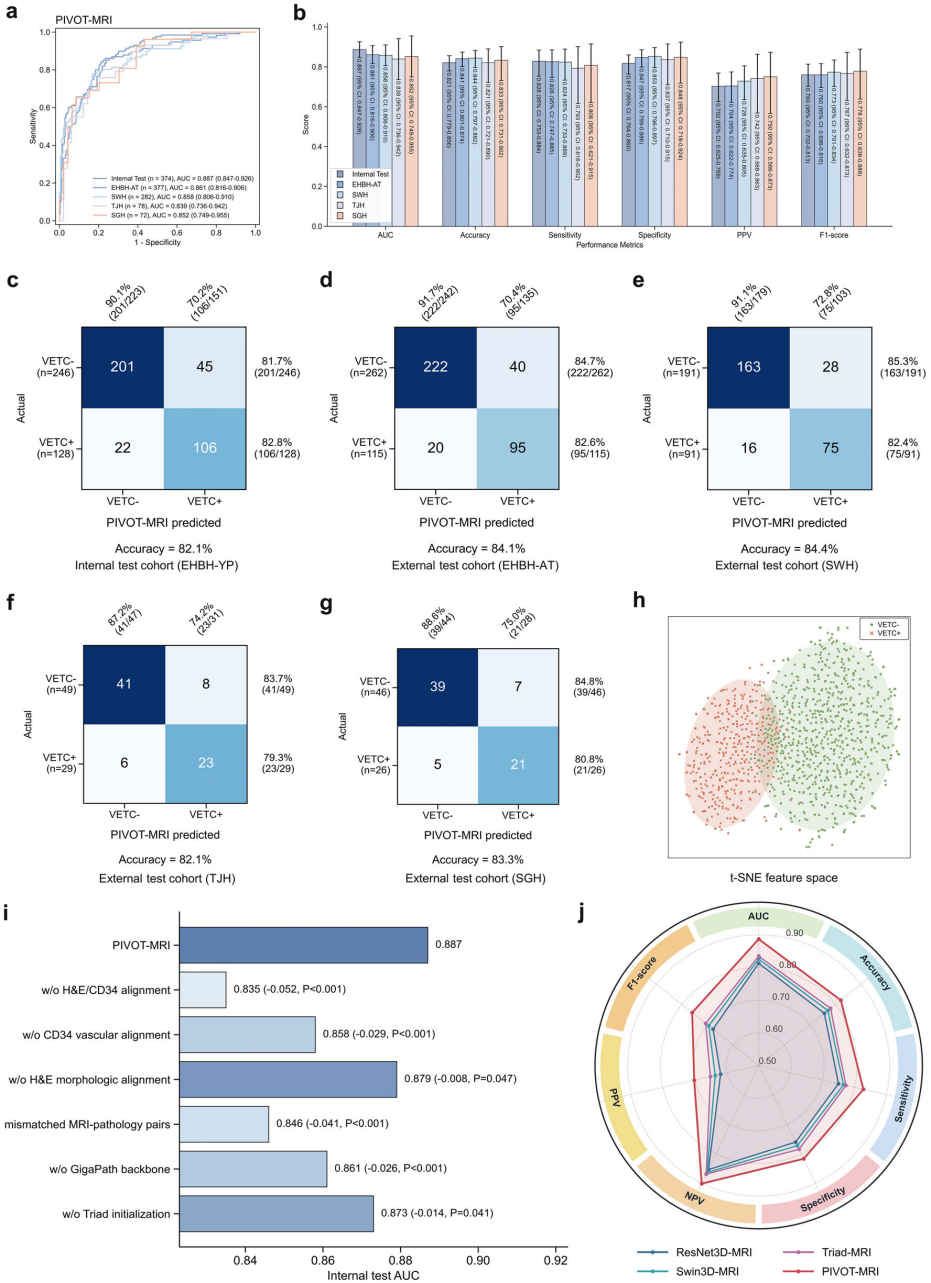
Multicenter diagnostic performance and ablation analysis of PIVOT‐MRI. (a) Receiver operating characteristic curves for the internal test cohort and four external validation cohorts. (b) AUC, accuracy, sensitivity, specificity, positive predictive value, and F1‐score with 95% confidence intervals. (c‐g) Confusion matrices for the internal test, EHBH‐AT, SWH, TJH, and SGH cohorts, respectively. Cells show patient counts and within‐reference‐class percentages; marginal annotations show class denominators, negative and positive predictive values, specificity, sensitivity, and overall accuracy. (h) t‐SNE projection of PIVOT‐MRI feature embeddings in the internal test cohort. (i) Internal‐test AUCs for the complete model and prespecified ablation variants. (j) Radar comparison of PIVOT‐MRI with MRI‐based deep learning models. AUC, area under the receiver operating characteristic curve; CI, confidence interval; NPV, negative predictive value; PPV, positive predictive value; t‐SNE, t‐distributed stochastic neighbor embedding; VETC, vessels that encapsulate tumor clusters.

Calibration and decision curve analyses were conducted separately in each validation cohort (Figure S1). Brier scores, LOWESS‐based integrated calibration indices, and calibration intercepts and slopes are reported in Table S4. PIVOT‐MRI generally provided greater net benefit than the treat‐all and treat‐none strategies across threshold probabilities of 0.10–0.60.

### 2.3 Contribution of Histopathologic Alignment to PIVOT‐MRI Performance

Ablation analyses showed that removal of both H&E‐ and CD34‐derived alignment losses reduced the internal‐test AUC from 0.887 to 0.835, with a corresponding decrease in mean external AUC from 0.853 to 0.797 (Main Table 3). Removal of CD34‐derived vascular alignment reduced the internal‐test AUC to 0.858 and the mean external AUC to 0.819, whereas removal of H&E‐derived morphologic alignment reduced the internal‐test AUC to 0.879 and the mean external AUC to 0.837. Random mismatching of paired MRI and pathology data reduced the internal‐test AUC to 0.846 and the mean external AUC to 0.810. Replacing the GigaPath backbone for pathology representation learning reduced the internal‐test AUC to 0.861 and the mean external AUC to 0.822, and removing Triad initialization from the MRI encoder reduced the internal‐test AUC to 0.873 and the mean external AUC to 0.832.

**TABLE 3:** Ablation Study of the PIVOT Model.

Model variant	Internal test	EHBH‐AT	SWH	TJH	SGH	*p* value*
PIVOT‐MRI	0.887 (0.847, 0.926)	0.861 (0.816, 0.906)	0.858 (0.806, 0.910)	0.839 (0.736, 0.942)	0.852 (0.749, 0.955)	/
w/o H&E/CD34 alignment	0.835 (0.788, 0.882)	0.812 (0.763, 0.861)	0.803 (0.745, 0.861)	0.780 (0.671, 0.891)	0.794 (0.685, 0.905)	<0.001
w/o CD34 vascular alignment	0.858 (0.814, 0.902)	0.836 (0.789, 0.883)	0.823 (0.767, 0.879)	0.801 (0.691, 0.911)	0.814 (0.705, 0.925)	<0.001
w/o H&E morphologic alignment	0.879 (0.838, 0.920)	0.854 (0.809, 0.899)	0.842 (0.789, 0.895)	0.819 (0.712, 0.926)	0.833 (0.725, 0.941)	0.047
mismatched MRI‐pathology pairs	0.846 (0.800, 0.892)	0.824 (0.776, 0.872)	0.814 (0.757, 0.871)	0.792 (0.682, 0.902)	0.809 (0.699, 0.919)	<0.001
w/o GigaPath backbone	0.861 (0.818, 0.904)	0.839 (0.793, 0.885)	0.828 (0.773, 0.883)	0.805 (0.697, 0.913)	0.817 (0.707, 0.927)	<0.001
w/o Triad initialization	0.873 (0.831, 0.915)	0.849 (0.804, 0.894)	0.837 (0.783, 0.891)	0.813 (0.705, 0.921)	0.829 (0.721, 0.937)	0.041

Note.—*p values compare AUCs between each model variant and PIVOT‐MRI in the internal test cohort using the paired DeLong test.

Paired MRI‐pathology embeddings had higher cosine similarity than randomly mismatched pairs in the internal test cohort. Mean cosine similarity was 0.67 for MRI‐H&E morphologic features and 0.71 for MRI‐CD34 vascular features, compared with 0.32 and 0.34 after random mismatch, respectively (both P < 0.001; Table S5). These paired‐vs.‐mismatched analyses support patient‐level correspondence between MRI‐derived and histopathologic features.

### 2.4 Performance of PIVOT‐Clinical and Comparison With Other Models

PIVOT‐Clinical, which incorporated PIVOT‐MRI score and preoperative clinical variables, achieved an internal AUC of 0.904 (95% CI, 0.867–0.941) and external AUCs ranging from 0.851 (95% CI, 0.752–0.950) to 0.884 (95% CI, 0.844–0.924). Compared with PIVOT‐MRI, PIVOT‐Clinical showed a significantly higher AUC in the internal test cohort (0.904 vs.0.887; p = 0.041) and a higher mean external AUC (0.867 vs.0.853). External Brier scores for PIVOT‐Clinical ranged from 0.137 to 0.154, and LOWESS‐based integrated calibration index values ranged from 0.062 to 0.112 (Figure S1 and Table S4). Across threshold probabilities of 0.10–0.60, PIVOT‐Clinical provided greater net benefit than PIVOT‐MRI throughout the internal, EHBH‐AT, and SWH cohorts and across most thresholds in TJH and SGH. The diagnostic performance, multivariable model, and predictor‐level explanations of PIVOT‐Clinical are shown in Figure 4a–f.

**FIGURE 4:**
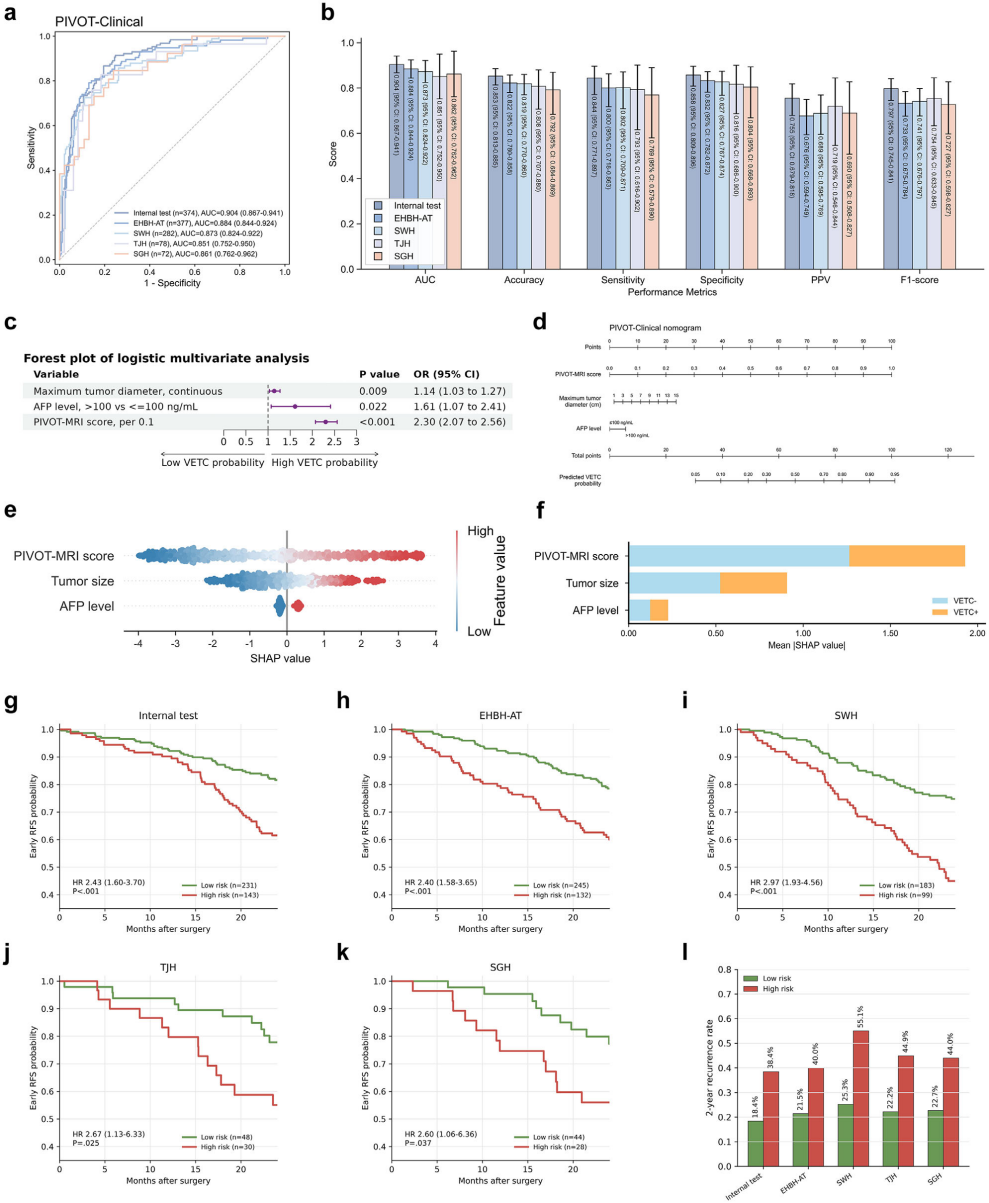
Clinical integration, diagnostic performance, interpretability, and recurrence association of PIVOT‐Clinical. (a) Receiver operating characteristic curves for the internal test cohort and four external validation cohorts. (b) AUC, accuracy, sensitivity, specificity, positive predictive value, and F1‐score with 95% confidence intervals. (c) Multivariable logistic regression model showing odds ratios and 95% confidence intervals for maximum tumor diameter, AFP level, and PIVOT‐MRI score. (d) Nomogram for estimating VETC probability. (e) SHAP summary plot showing the direction and magnitude of predictor contributions. (f) Mean absolute SHAP values by VETC group. (g–k) Kaplan‐Meier curves for early recurrence‐free survival in the internal test, EHBH‐AT, SWH, TJH, and SGH cohorts, respectively. (l) Two‐year recurrence rates from the corresponding Kaplan‐Meier analyses in the high‐ and low‐risk groups. The development‐cohort VETC classification cutoff was applied unchanged in all validation cohorts. AFP, alpha‐fetoprotein; AUC, area under the receiver operating characteristic curve; CI, confidence interval; RFS, recurrence‐free survival; SHAP, Shapley additive explanations; VETC, vessels that encapsulate tumor clusters.

The clinical‐only model, which included maximum tumor diameter and AFP, achieved an internal‐test AUC of 0.670 and external AUCs of 0.649, 0.651, 0.621, and 0.631 in EHBH‐AT, SWH, TJH, and SGH, respectively. PIVOT‐MRI showed significantly higher AUCs across all validation cohorts (Main Table 4 and Table S3).

Compared with MRI‐based deep learning models, PIVOT‐MRI had a higher internal‐test AUC than ResNet3D‐MRI (0.887 vs.0.813; P < 0.001), Swin3D‐MRI (0.887 vs.0.842; P < 0.001), and Triad‐MRI (0.887 vs.0.835; P < 0.001; Main Table 4). Across external cohorts, the AUC ranges were 0.758 (95% CI, 0.648–0.868) to 0.789 (95% CI, 0.729–0.849) for ResNet3D‐MRI, 0.785 (95% CI, 0.675–0.895) to 0.816 (95% CI, 0.767–0.865) for Swin3D‐MRI, and 0.781 (95% CI, 0.671–0.891) to 0.812 (95% CI, 0.763–0.861) for Triad‐MRI, compared with 0.839 (95% CI, 0.736–0.942) to 0.861 (95% CI, 0.816–0.906) for PIVOT‐MRI. Among histopathologic models, GigaPath‐H&E achieved an internal AUC of 0.916 (95% CI, 0.881–0.951), and external AUCs ranging from 0.871 (95% CI, 0.778–0.966) to 0.895 (95% CI, 0.858–0.932; internal‐test P < 0.001 vs. PIVOT‐MRI; Table S6). GigaPath‐CD34 achieved an internal AUC of 0.973 (95% CI, 0.955–0.991) and external AUCs ranging from 0.947 (95% CI, 0.899–0.995) to 0.961 (95% CI, 0.939–0.983; internal‐test P < 0.001 vs.PIVOT‐MRI). GigaPath‐H&E and GigaPath‐CD34 used postoperative tissue images.

**TABLE 4:** Diagnostic Performance of the Clinical‐Only Model, MRI‐Based Deep Learning Models, PIVOT‐MRI, and PIVOT‐Clinical.

Model	EHBH‐YP internal test AUC	EHBH‐AT AUC	SWH AUC	TJH AUC	SGH AUC	*p* value*
ResNet3D‐MRI	0.813 (0.764, 0.862)	0.779 (0.727, 0.831)	0.789 (0.729, 0.849)	0.758 (0.648, 0.868)	0.766 (0.656, 0.876)	<0.001
Swin3D‐MRI	0.842 (0.796, 0.888)	0.816 (0.767, 0.865)	0.806 (0.748, 0.864)	0.785 (0.675, 0.895)	0.796 (0.686, 0.906)	<0.001
Triad‐MRI	0.835 (0.788, 0.882)	0.812 (0.763, 0.861)	0.803 (0.745, 0.861)	0.781 (0.671, 0.891)	0.795 (0.685, 0.905)	<0.001
Clinical‐only	0.670 (0.611, 0.725)	0.649 (0.587, 0.707)	0.651 (0.578, 0.720)	0.621 (0.486, 0.749)	0.631 (0.492, 0.767)	<0.001
PIVOT‐MRI	0.887 (0.847, 0.926)	0.861 (0.816, 0.906)	0.858 (0.806, 0.910)	0.839 (0.736, 0.942)	0.852 (0.749, 0.955)	/
PIVOT‐Clinical	0.904 (0.867, 0.941)	0.884 (0.844, 0.924)	0.873 (0.824, 0.922)	0.851 (0.752, 0.950)	0.861 (0.762, 0.962)	0.041

Note.—*p values compare AUCs between each model and PIVOT‐MRI in the internal test cohort using the paired DeLong test.

### 2.5 PIVOT‐Clinical Stratification of Early Recurrence Risk

In unadjusted analyses, Kaplan‐Meier analysis using the fixed PIVOT‐Clinical cutoff showed significant differences in early recurrence‐free survival between the high‐ and low‐risk groups across validation cohorts (Table S7). In the internal test cohort, the 2‐year recurrence rate was higher in the high‐risk group than in the low‐risk group (38.4% vs.18.4%; HR, 2.43; 95% CI, 1.60–3.70; log‐rank P < 0.001). Significant between‐group differences were also observed in each external cohort, with higher 2‐year recurrence rates in the high‐risk group than in the low‐risk group in EHBH‐AT (40.0% vs.21.5%; HR, 2.40; 95% CI, 1.58–3.65; P < 0.001), SWH (55.1% vs.25.3%; HR, 2.97; 95% CI, 1.93–4.56; P < 0.001), TJH (44.9% vs.22.2%; HR, 2.67; 95% CI, 1.13–6.33; p = 0.025), and SGH (44.0% vs.22.7%; HR, 2.60; 95% CI, 1.06–6.36; p = 0.037). The fixed cutoff consistently stratified patients into groups with different 2‐year recurrence rates across centers. Cohort‐specific recurrence‐free survival curves and 2‐year recurrence rates are shown in Figure 4g–l.

### 2.6 PIVOT Visualization and Biological Interpretation

SHAP analysis was used to examine the relative contribution of PIVOT‐MRI score and preoperative clinical variables in PIVOT‐Clinical. The SHAP summary and feature‐importance plots showed that the PIVOT‐MRI score contributed most to model output, followed by maximum tumor diameter and AFP level, indicating that the imaging‐derived score accounted for most of the model‐predicted VETC risk. Individual SHAP explanation plots further illustrated patient‐level prediction patterns, with higher PIVOT‐MRI score increasing the predicted probability of VETC positivity.

MRI activation maps were generated to visualize image regions contributing to PIVOT‐MRI predictions. High‐score cases most frequently showed activation in areas of intratumoral enhancement heterogeneity, the tumor margin, and the peritumoral parenchyma. In lower‐score cases, activation was less concentrated along irregular enhancing tumor margins and was more diffusely distributed within the tumor region. These spatial patterns provided image‐level explanations consistent with radiologic manifestations of heterogeneous vascularity and invasive tumor morphology.

#### 2.6.1 Radiotranscriptomic Immune Microenvironment Analysis

The biological context of PIVOT‐derived VETC risk was explored in the TCGA‐LIHC subset with matched MRI and RNA‐sequencing data (n = 29). Using the cutoff derived from the development cohort, 9 patients were classified into the high‐score group and 20 into the low‐score group. Differential expression analysis identified 496 genes that differed between high‐ and low‐PIVOT‐MRI‐score tumors at an FDR threshold of 0.05. Exploratory signature analysis showed positive correlations of PIVOT‐MRI score with hypoxia‐angiogenesis, proliferative, vascular endothelial, immune‐checkpoint macrophage, and M2 macrophage signatures, and negative correlations with hepatic metabolism, cytochrome P450/xenobiotic metabolism, bile acid metabolism, and PPAR signaling signatures (Table S8). Immune and stromal abundance scores were estimated from bulk RNA‐sequencing data using MCP‐counter. Monocytic‐lineage abundance was nominally higher in the high‐score group (median score, 3.40 vs.2.59; p = 0.007) but did not meet the FDR threshold (FDR = 0.071); cytotoxic lymphocyte and NK‐cell abundance scores also did not differ significantly between groups. Overall, these exploratory radiotranscriptomic analyses identified associations between higher PIVOT‐MRI scores and angiogenesis‐related, endothelial, and proliferative transcriptional programs, providing hypothesis‐generating biological context for the imaging phenotype.

#### 2.6.2 Gene Pathway Enrichment Analysis

To investigate transcriptomic programs associated with PIVOT‐derived VETC risk, gene set enrichment analysis was performed using GO biological process and KEGG pathway databases in the TCGA‐LIHC MRI‐RNA subset. The pathway analyses were based on a differential expression contrast of high‐ vs. low‐PIVOT‐MRI‐score tumors. Pathways shown in the activated panel correspond to gene sets enriched among genes upregulated in the high‐score group, whereas pathways shown in the suppressed panel correspond to gene sets relatively more active in the low‐score group.

For GO biological processes, high‐score tumors showed enrichment of cell‐cycle phase transition, mitotic cell‐cycle transition, cell‐substrate adhesion, TGF‐beta response, and extracellular matrix organization. For KEGG pathways, high‐score tumors showed enrichment of cell cycle, proteoglycans in cancer, focal adhesion, and PI3K‐Akt signaling pathways. In contrast, low‐score tumors showed relative enrichment of hepatic metabolic programs, including fatty acid degradation, primary bile acid biosynthesis, PPAR signaling, and cytochrome P450/xenobiotic metabolism pathways. These exploratory results were consistent with greater angiogenesis‐related, endothelial, and proliferative transcriptional activity at higher PIVOT‐MRI scores and greater hepatocellular metabolic activity at lower scores. Representative activation maps and the exploratory radiotranscriptomic analyses are shown in Figure 5.

**FIGURE 5:**
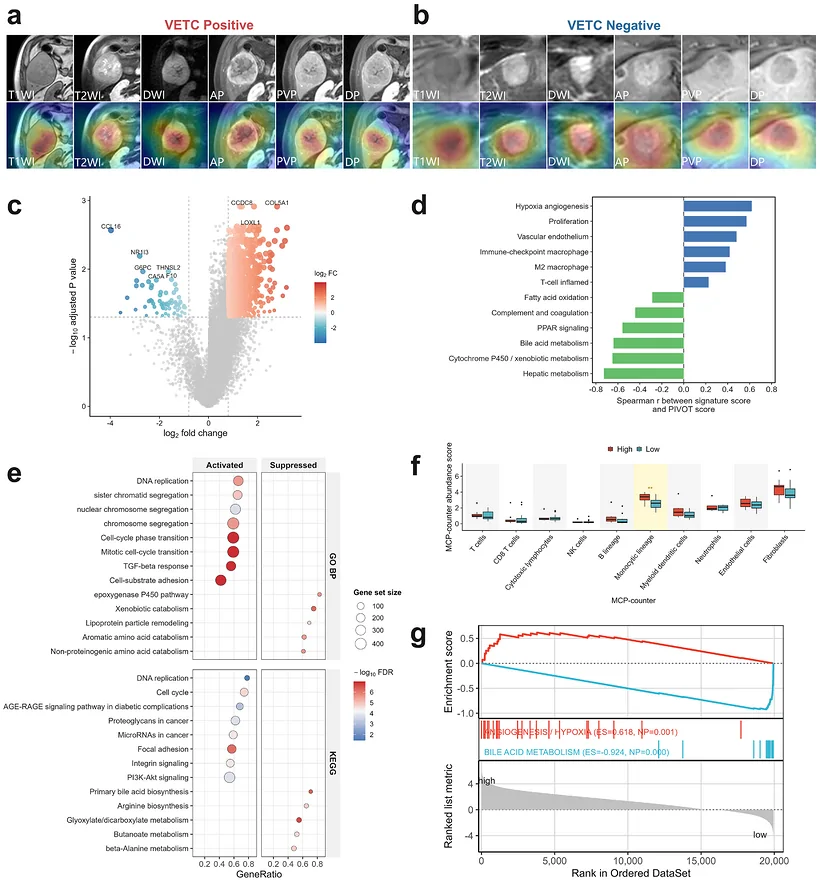
MRI activation patterns and exploratory radiotranscriptomic associations of the PIVOT‐MRI score. (a, b) Representative six‐sequence MRI images and corresponding activation maps from VETC‐positive and VETC‐negative tumors. (c) Differential‐expression volcano plot comparing high‐ and low‐PIVOT‐MRI‐score tumors in the TCGA‐LIHC MRI‐RNA subset. (d) Spearman correlations between the PIVOT‐MRI score and prespecified biological signature scores. (e) Gene Ontology biological‐process and KEGG pathway enrichment among programs activated at higher scores or relatively enriched at lower scores. (f) MCP‐counter estimates of immune and stromal‐cell abundance in high‐ and low‐score groups. (g) Gene set enrichment plots for hypoxia/angiogenesis and bile acid metabolism. All molecular analyses were exploratory and were performed in 29 patients with matched pretreatment MRI and RNA‐sequencing data. AP, arterial phase; DP, delayed phase; DWI, diffusion‐weighted imaging; GSEA, gene set enrichment analysis; KEGG, Kyoto Encyclopedia of Genes and Genomes; PVP, portal venous phase; TCGA‐LIHC, The Cancer Genome Atlas Liver Hepatocellular Carcinoma; VETC, vessels that encapsulate tumor clusters.

## 3 Discussion

VETC is an aggressive vascular phenotype of HCC associated with hematogenous dissemination and early recurrence, yet its definitive assessment generally depends on CD34 immunohistochemistry of resected tissue [6, 7, 9, 10]. In this multicenter study of 2052 patients, PIVOT‐MRI achieved AUCs of 0.839–0.861 across four external cohorts. PIVOT‐Clinical, which combined the MRI score with tumor size and AFP, achieved an AUC of 0.904 internally and AUCs of 0.851–0.884 externally. During model development, paired H&E and CD34 histopathology images from resected specimens were used to guide MRI representation learning. For clinical application, PIVOT‐Clinical provides a patient‐level probability of VETC based solely on preoperative MRI, tumor size, and AFP, thereby enabling preoperative assessment of a vascular pattern associated with tumor dissemination [6].

Previous CT and MRI studies have estimated VETC using qualitative imaging features, radiomics, and deep learning models [10, 11, 12, 13, 14, 15, 16]. These studies established that VETC has detectable imaging correlates. Qualitative features are readily interpretable but depend on reader assessment, whereas most quantitative models use the binary pathologic VETC label as supervision and do not incorporate image‐level information from the corresponding histopathology slides. Yu et al. subsequently combined MRI‐ and H&E‐derived scores for outcome stratification, an approach that still required tissue‐derived information at application [14]. More broadly, radiology‐pathology alignment has shown that histology can guide image representation learning while diagnosis remains based on radiologic imaging [24]. PIVOT applied this principle to CD34‐defined VETC by using H&E and CD34 only as development‐stage references. This distinction is clinically relevant because HCC diagnosis and treatment planning often proceed without tissue confirmation [2, 3, 10]. It permits histopathology obtained after surgery to strengthen a model intended for use before surgery, without altering the inputs required from the patient.

The ablation and mismatch analyses provided complementary evidence that histopathology‐derived alignment and correct patient‐level pairing contributed to model performance. Removing H&E‐ and CD34‐based alignment reduced performance in internal and external testing, and the larger decrease after removal of CD34 alignment was consistent with the central role of CD34 immunohistochemistry in VETC assessment [6, 7]. Deliberately mismatching MRI and histopathology images also reduced performance, whereas correctly paired data retained stronger cross‐modal correspondence. Together, these analyses indicate that H&E‐ and CD34‐derived alignment targets and correct patient‐level pairing both contributed to model performance. However, the pathology‐informed alignment should not be interpreted as transferring purely label‐independent histopathologic information to MRI. Because both pathology reference models were fine‐tuned using the VETC label, the alignment strategy also has features of teacher‐student representation learning and provides additional supervised constraints on MRI representation learning. Therefore, the current experiments do not fully distinguish whether the observed performance gain arises from pathology‐specific information, additional supervision, or regularization.

PIVOT‐Clinical combined the MRI score with tumor size and AFP, all of which are available before surgery. It showed generally acceptable calibration and greater net benefit than PIVOT‐MRI across most intermediate decision thresholds, suggesting potential decision‐analytic utility under the prespecified clinical scenario. However, this finding should not be interpreted as evidence of established clinical utility, because whether PIVOT‐guided management improves patient outcomes has not been prospectively evaluated. Automated segmentation showed stable performance across centers, supporting its use as the localization component of the PIVOT pipeline. Across all validation cohorts, higher PIVOT‐Clinical scores were consistently associated with higher 2‐year recurrence rates in unadjusted analyses, in keeping with the established association of VETC with aggressive dissemination and early recurrence [6, 7, 9, 10]. For patients selected for curative‐intent resection, these findings support further evaluation of PIVOT‐Clinical for preoperative early‐recurrence risk stratification and could define a target population for prospective studies of postoperative recurrence surveillance. Separately, the retrospective association between tissue‐defined VETC status and differential sorafenib benefit [8] provides a rationale for evaluating PIVOT‐Clinical as a candidate noninvasive stratification marker in prospective sorafenib studies.

Within the fitted PIVOT‐Clinical model, the PIVOT‐MRI score had the largest SHAP contribution, indicating that the final probability estimate was primarily imaging‐driven. Activation maps highlighted the tumor, tumor margin, and peritumoral liver, regions routinely examined on preoperative imaging for aggressive HCC phenotypes [10, 11, 12, 13, 14, 15, 16]. These activation maps should be interpreted as model‐visualization tools rather than evidence that the highlighted regions are biologically causal or specifically represent VETC‐associated vascular structures. In the exploratory TCGA‐LIHC cohort, higher PIVOT‐MRI scores were associated with hypoxia‐angiogenesis, endothelial, and proliferative programs, whereas lower scores were associated with hepatocellular metabolic programs. The vascular and hypoxia‐related pattern was concordant with the angiogenic biology described for VETC [6, 7, 9]. Given the small sample size and the use of bulk RNA sequencing, these associations provide hypothesis‐generating biological context for the imaging phenotype rather than evidence that PIVOT‐MRI specifically reflects endothelial cells or VETC‐associated vascular structures. Accordingly, the spatial and transcriptomic findings should be regarded as exploratory observations that motivate further spatially matched biological validation rather than mechanistic validation of PIVOT.

Several limitations should be considered. The retrospective cohorts comprised patients with HCC who underwent curative resection, which may have introduced selection and spectrum bias. Prospective validation in the intended surgical population, as well as evaluation in patients with unresectable or advanced HCC, is therefore needed to establish the generalizability of PIVOT beyond the current study population. In addition, the recurrence analyses were unadjusted and were intended to assess risk stratification rather than establish independent prognostic value. The prognostic contribution of PIVOT‐MRI independent of established clinicopathologic factors therefore remains to be determined. Future studies incorporating different surgical strategies should also assess whether the association between preoperative VETC estimates and postoperative recurrence varies across operative approaches. Although external validation encompassed multiple centers, scanners, and acquisition protocols, all participating institutions were in China; geographically broader validation is warranted to establish applicability across different liver disease backgrounds and care settings. The current PIVOT‐MRI framework also requires all six MRI sequences, and its performance when individual sequences are unavailable or when acquisition protocols differ substantially from those represented in the training data was not evaluated. Future work should assess robustness to missing sequences and develop flexible modeling strategies for incomplete MRI protocols to improve applicability in routine clinical practice. Because VETC may exhibit spatial heterogeneity, incomplete spatial sampling of CD34‐stained sections may introduce uncertainty into the pathological reference labels and potentially affect model development and performance estimation. Finally, the radiotranscriptomic cohort was modest, and these findings should be regarded as exploratory biological context for the imaging score. Prospective evaluation in broader populations and larger matched imaging‐omics cohorts will be important for further clinical validation.

In conclusion, PIVOT used postoperative histopathology to inform learning from preoperative MRI while retaining an MRI‐based assessment pathway for clinical application. Its consistent performance across four external cohorts supports further clinical validation of preoperative VETC assessment in patients selected for curative‐intent resection, while the observed recurrence associations motivate prospective evaluation of PIVOT‐Clinical for early‐recurrence risk stratification in this setting.

## 4 Experimental Section

### 4.1 Study Design and Population

A total of 2052 patients with HCC were retrospectively enrolled from five medical centers between January 2016 and January 2025. Patients from EHBH‐YP were randomly divided into the training cohort (n = 869) and internal test cohort (n = 374). Independent external validation cohorts were obtained from EHBH‐AT (n = 377), SWH (n = 282), TJH (n = 78), and SGH (n = 72). The patient selection process is shown in Figure 2 and Methods S1, and cohort distribution and VETC prevalence are summarized in Main Table 1.

This study was approved by the institutional review boards of the participating medical centers and was conducted in accordance with the Declaration of Helsinki. In accordance with institutional regulations and the retrospective nature of the study, the requirement for written informed consent was waived. All imaging, pathology, and clinical data were de‐identified before model development and validation.

The inclusion criteria were as follows: (i) standardized liver MRI performed within 2 months prior to surgery; (ii) curative‐intent hepatic resection with histopathologic confirmation of HCC; (iii) definitive VETC status determined by postoperative pathology with CD34 immunohistochemistry; (iv) availability of diagnostic‐quality MRI, digitized hematoxylin and eosin (H&E)‐stained whole‐slide images, and digitized CD34 immunohistochemistry slides; and (v) availability of complete preoperative clinical data. The exclusion criteria included: (i) prior locoregional or systemic antitumor treatment before MRI, including transarterial chemoembolization, ablation, radiotherapy, systemic therapy, or other antitumor treatments; (ii) distant metastasis, macrovascular invasion, tumor thrombus, or other concurrent malignant tumors; (iii) inadequate MRI or digitized pathology image quality for analysis; (iv) unavailable CD34 immunohistochemistry or insufficient tumor‐containing CD34‐stained tissue for reliable VETC assessment; (v) missing essential clinicopathologic data; or (vi) failure to link imaging, pathology, and clinical records.

### 4.2 Histopathologic Assessment of VETC

VETC was defined as the presence of a cobweb‐like vascular network composed of CD34‐positive endothelial cells encapsulating tumor cell clusters [6]. The extent of VETC was semi‐quantitatively evaluated from 0% to 100% in 5% increments. Tumors with a VETC proportion ≥5% were classified as VETC‐positive, whereas those with <5% were considered VETC‐negative.

VETC status was determined on postoperative CD34 immunohistochemistry slides and served as the reference label for model development and validation. At each participating center, VETC was independently assessed by two pathologists with more than 5 years of experience in liver pathology, who were blinded to model predictions and recurrence outcomes. When more than one tumor‐containing CD34‐stained slide was available from the same tumor, the VETC proportion was assessed separately on each slide, and the highest slide‐level proportion was used as the final case‐level VETC proportion to determine the binary VETC status. The two pathologists performed their initial assessments independently. Disagreements were resolved through joint review and consensus; any unresolved discrepancies were adjudicated by a third senior pathologist with more than 10 years of experience in liver pathology. H&E‐stained whole‐slide images were used to derive morphologic histopathologic representations, and CD34 immunohistochemistry was used to define the reference label and derive vascular histopathologic representations.

### 4.3 Follow‐Up Data

Follow‐up began on the day after surgery, and survival time was calculated in months. Follow‐up data were mainly collected from electronic medical records and telephone contact. During the first two years after surgery, patients underwent contrast‐enhanced upper abdominal computed tomography or MRI every 3 months, together with routine laboratory tests, including serum AFP and liver function tests. The follow‐up period was capped at two years for the present analysis.

Early recurrence‐free survival was defined as the time from surgery to the first documented recurrence within two years. Recurrence events included local recurrence, intrahepatic distant recurrence, and extrahepatic metastasis. Patients without documented recurrence within 2 years were censored at the last available follow‐up or at 2 years after surgery, whichever came first.

### 4.4 MRI Examination and Preprocessing

MRI data were acquired using 1.5 or 3.0T scanners at five centers. The MRI protocol included fat‐suppressed T2‐weighted imaging (T2WI), T1‐weighted imaging (T1WI), diffusion‐weighted imaging (DWI), and contrast‐enhanced imaging in the arterial phase (AP), portal venous phase (PVP), and delayed phase (DP). Detailed acquisition parameters are listed in Methods S2 and Table S1.

All images underwent standardized preprocessing, including format conversion, voxel resampling, intensity normalization, and rigid registration to PVP images to ensure inter‐center consistency. For patients with multiple lesions, the largest lesion by maximum diameter was selected as the index tumor. Tumor segmentation maps and sequence‐specific images were registered to the PVP reference space for PIVOT assessment.

### 4.5 MRI Tumor Annotation

Manual annotation was performed to provide reference tumor localization for automated segmentation model development and validation. For each patient, the index tumor was identified on dynamic contrast‐enhanced MRI with reference to all available sequences and clinical reports. Tumor contours were delineated on PVP images by trained radiologists and then reviewed by a senior abdominal radiologist. Liver contours were annotated on the same reference images to support joint liver‐tumor segmentation and to constrain subsequent automated tumor localization. For multifocal HCC, the lesion with the largest maximum diameter was annotated as the index tumor. Disagreements regarding lesion selection or tumor boundary were resolved by consensus.

All manual annotations were completed before model evaluation and were not informed by PIVOT predictions. After rigid registration, the PVP‐based tumor contours were transferred to other MRI sequences and visually checked for anatomic alignment. Cases with substantial motion artifact, failed registration, incomplete tumor coverage, or uncertain lesion matching underwent additional radiologic review. Manual radiologic imaging features were not used as model inputs or comparator variables in the present analysis.

### 4.6 Automated MRI Tumor Segmentation

The manually annotated liver and index‐tumor contours served as the reference standard for evaluating automated tumor localization. A 3D nnU‐Net [25] model was trained on PVP images from EHBH‐YP for joint liver‐tumor segmentation and subsequently validated on datasets from four independent centers. To reduce false‐positive localizations, tumor segmentations were constrained within the liver region. Each validation patient contributed one index tumor. Successful automated localization was defined at the patient level as a DSC ≥0.80 between the automatically segmented tumor and the manually delineated index‐tumor reference. For patients with multifocal HCC, the lesion with the largest maximum diameter was predefined as the index tumor. When multiple candidate tumor lesions were detected automatically, the candidate corresponding to the predefined index tumor was selected for evaluation.

For evaluation of the automated localization and segmentation component itself, the automatically generated tumor masks were evaluated without manual correction or refinement. Automated segmentation results were subject to spot‐check manual review for quality control rather than case‐by‐case manual confirmation. Cases that did not meet the predefined localization‐success criterion were classified as automated localization failures. Importantly, these cases were not excluded from the subsequent PIVOT performance analysis. Instead, consistent with the intended real‐world clinical workflow, a manual fallback procedure was applied, whereby a radiologist corrected the erroneous tumor segmentation, after which the case proceeded through the same downstream PIVOT analysis as successfully automated cases.

After registration to the PVP reference space, multiparametric MRI data from T1WI, T2WI, DWI, AP, PVP, and DP images were prepared for model development and validation. Segmentation performance was evaluated using the DSC by comparing automated segmentations with manual annotations. Liver DSC was calculated in all validation patients, whereas index‐tumor DSC was calculated among patients with successful automated index‐tumor localization. Additional details are provided in Methods S2.

### 4.7 Histopathologic Slide Preprocessing

All available H&E‐stained and CD34 immunohistochemistry slides from patients with resectable HCC across five centers were collected. Slides were digitized at 40× magnification to generate WSIs. Two experienced pathologists independently reviewed all slides, and those with significant artifacts, including folding, tearing, severe blur, or staining failure, were excluded. Cohort assignment was performed at the patient level.

To reduce inter‐center staining variability, stain normalization was applied before computational processing. Following commonly used weakly supervised WSI pipelines, foreground tissue regions were segmented automatically, and background, folds, severe blur, staining artifacts, and regions with insufficient tissue were excluded. Non‐overlapping 256 × 256‐pixel tiles were then extracted from all eligible tissue regions. Tile extraction was performed independently of VETC status and without manual selection of VETC‐suspicious regions. Tile coordinates were retained for subsequent whole‐slide representation learning. Technical details of tissue segmentation, quality control, tiling, and feature extraction are provided in Methods S3.

### 4.8 PIVOT Model Development

PIVOT was designed to estimate VETC status before surgery in patients with HCC using pretreatment MRI. During model development, paired postoperative H&E WSIs and CD34 immunohistochemistry images from the same tumors provided morphologic and vascular histopathologic information to guide MRI representation learning. This strategy enabled histopathologic knowledge transfer to the MRI model during training, while PIVOT assessment in the intended preoperative decision setting was performed from routine pretreatment MRI [24].

#### 4.8.1 Histopathologic Model Development

Two stain‐specific histopathologic models were developed to separately characterize H&E‐derived tumor morphology and CD34‐derived vascular organization. H&E slides show histologic tumor architecture, cellular morphology, and the tumor‐stromal microenvironment, whereas CD34 immunohistochemistry highlights the vascular encasement pattern used for VETC assessment. Both models used Prov‐GigaPath, a whole‐slide pathology foundation model composed of a tile encoder and a WSI‐level slide encoder [26, 27]. Foreground tissue tiles retained after preprocessing were encoded and passed with their spatial coordinates to the slide encoder, which aggregated the spatially indexed tile embeddings into slide‐level embeddings. For each stain, slide‐level embeddings from all eligible slides of the same patient were summarized by a gated attention‐based patient‐level aggregation module to obtain a case‐level histopathologic embedding, and a lightweight prediction head estimated patient‐level VETC status [19, 28, 29]. The H&E and CD34 models were initialized from the same Prov‐GigaPath pretrained weights and fine‐tuned with weak supervision using the CD34‐defined patient‐level VETC status.

#### 4.8.2 MRI Model Development

The MRI component used tumor‐centered volumes from six registered MRI sequences, including T1WI, T2WI, DWI, AP, PVP, and DP. The 3D MRI encoder was initialized with Triad‐SwinB‐SimMIM, a large‐scale pretrained 3D MRI foundation model [30]. Each registered MRI sequence was encoded separately with shared Triad‐SwinB weights. Multiscale backbone features were globally pooled, concatenated, and projected into a 768‐dimensional sequence embedding, after which a sequence‐specific lightweight adapter was applied to account for differences in contrast weighting and acquisition characteristics. Each adapted representation was combined with a learnable sequence‐type embedding and treated as an MRI sequence token. A learnable VETC classification token was appended to the six MRI sequence tokens, and a lightweight sequence‐token transformer modeled inter‐sequence relationships and updated the VETC classification token. The updated VETC classification token was used for VETC prediction and was aligned in the embedding space with morphologic and vascular histopathologic embeddings for pathology‐guided alignment. Implementation‐level details are provided in Methods S4.

#### 4.8.3 MRI‐Pathology Embedding‐Space Alignment and Loss Function

After histopathologic fine‐tuning, the H&E and CD34 models were fixed and used to guide MRI model training. Because both histopathologic reference models were fine‐tuned using the CD34‐defined VETC label, the resulting embeddings should be regarded as VETC‐informed, pathology‐derived representations rather than label‐independent histopathologic features. Given that both VETC status and MRI assessment were defined at the case level, the updated VETC classification token was aligned with patient‐level histopathologic embeddings. During PIVOT development, the updated VETC classification token was aligned with the corresponding H&E‐derived morphologic embedding and CD34‐derived vascular embedding. The VETC prediction head was optimized using the CD34‐defined VETC status. The CD34‐derived vascular embedding was used as an auxiliary embedding‐space target, with its contribution controlled by a prespecified alignment‐loss weight. The contribution of CD34‐derived alignment was further evaluated in ablation analyses by removing the vascular alignment term and by randomly mismatching MRI‐pathology pairs. The two alignment losses were defined using scaled cosine error:

$$L_{morph}={\left[1-\cos\left({R_{i}}^{morph},\;v_{i}\right)\right]}^{\gamma }$$

$$L_{vasc}={\left[1-\cos\left({R_{i}}^{vasc},\;w_{i}\right)\right]}^{\gamma }$$

where *r_i_^moprh^* and *r_i_^vasc^* denote the updated MRI‐derived VETC classification token in the morphologic and vascular embedding spaces, respectively; *v_i_* denotes the patient‐level morphologic embedding derived from H&E WSIs; *w_i_* denotes the patient‐level vascular embedding derived from CD34 immunohistochemistry WSIs; *cos(·,·)* denotes cosine similarity; and *γ* denotes the scaling factor. The total loss combined VETC classification loss with the two embedding‐space alignment losses:

$$L_{total}=L_{cls}+\lambda_{morph}L_{morph}+\lambda_{vasc}L_{vasc}$$

The scaling factor was set to *γ = 3*. Because CD34 immunohistochemistry directly delineates the vascular architecture used for VETC assessment, whereas H&E WSIs provide broader morphologic context, the vascular alignment term was assigned a moderately higher prespecified weight than the morphologic alignment term (*λ_vasc_* = 0.10 and *λ_morph_* = 0.05). These weights were fixed before model evaluation and were not tuned on the internal test or external validation cohorts. The predefined sensitivity analysis is reported in Table S9.

Gradients were not propagated into the histopathologic models during MRI model development.

#### 4.8.4 Training Strategy and Clinical Inference

Model training was performed in the training cohort, and final model selection was based on validation AUC for VETC prediction. Following histopathologic model fine‐tuning, the H&E and CD34 models were fixed. The MRI model was first trained on paired MRI‐pathology cases using only the two embedding‐space alignment losses; subsequently, VETC classification loss was added, and the MRI model was jointly optimized for VETC prediction and pathology‐guided alignment. During MRI model development, the pretrained Triad encoder was initially frozen while newly introduced sequence‐adaptation, sequence‐token transformer, embedding‐alignment, and prediction modules were trained; the final Swin Transformer stage was then fine‐tuned with a lower learning rate, with earlier encoder stages kept frozen. After training, the prediction pathway used for clinical evaluation consisted of the MRI component, sequence‐token transformer, and VETC prediction head. In the preoperative clinical setting, PIVOT scoring was based on routinely available pretreatment MRI. Detailed architecture, optimization, loss functions, and fine‐tuning procedures are provided in Methods S4. The detailed PIVOT architecture, staged optimization, and MRI‐only inference pathway are shown in Figure 6.

**FIGURE 6:**
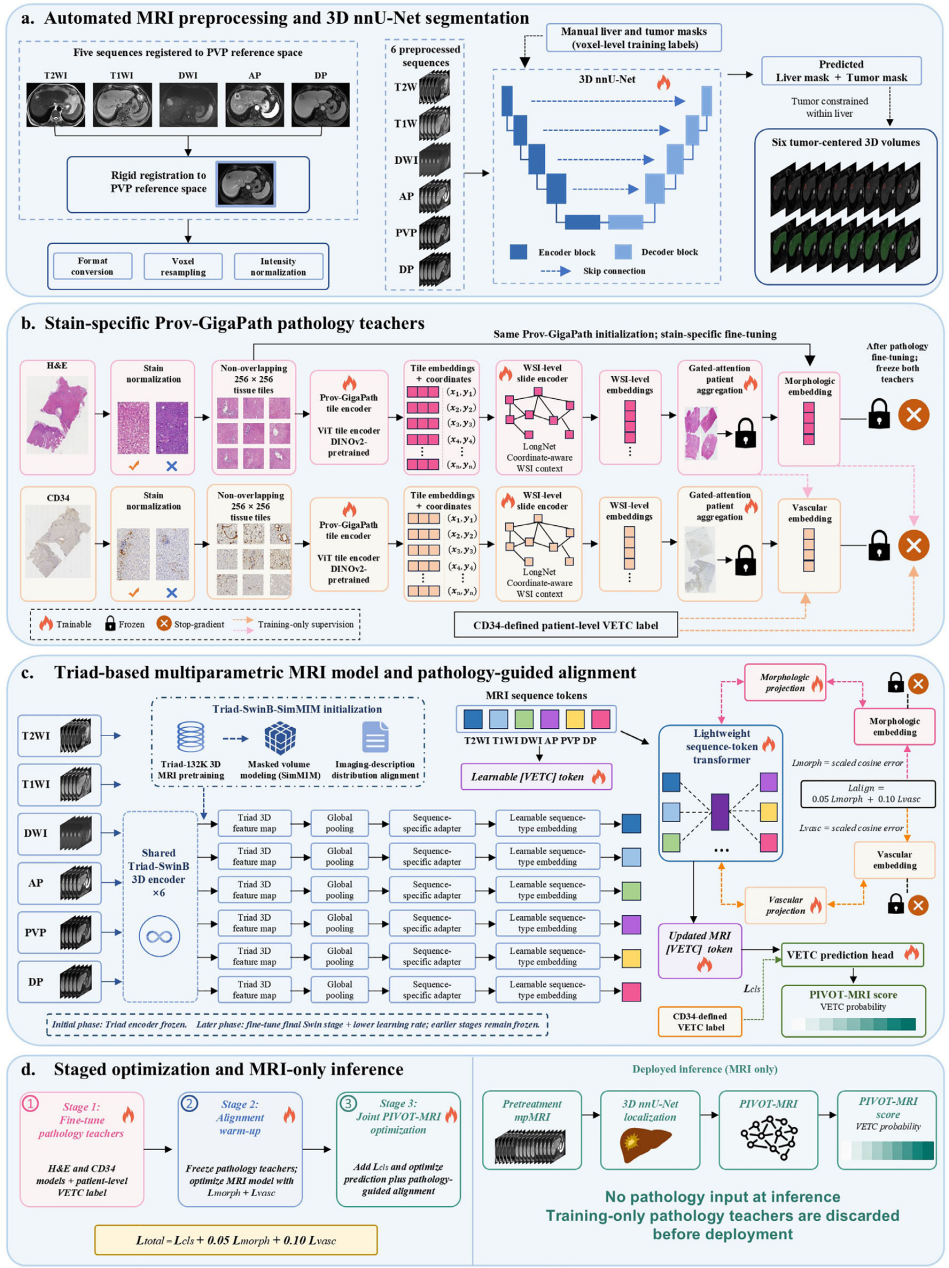
Detailed PIVOT architecture, staged optimization, and MRI‐only inference. (a) Six MRI sequences were registered to portal venous phase space, preprocessed, and passed through a 3D nnU‐Net for liver and index‐tumor localization before generation of tumor‐centered volumes. (b) Stain‐specific Prov‐GigaPath pathology teachers generated patient‐level H&E morphologic and CD34 vascular embeddings and were frozen after fine‐tuning. (c) A shared Triad‐SwinB encoder transformed the six MRI volumes into sequence tokens; a lightweight sequence‐token transformer updated the learnable VETC token, which supported classification and separate morphologic and vascular projections for training‐time representation alignment. (d) Staged optimization comprised pathology‐teacher fine‐tuning, alignment warm‐up, and joint PIVOT‐MRI optimization. The total loss was Lcls + 0.05Lmorph + 0.10Lvasc. At deployment, the pathology teachers and alignment branches were discarded, and inference required only preoperative MRI. Flame symbols indicate trainable components, lock symbols indicate frozen components, crossed‐circle symbols indicate stop‐gradient operations, and colored dashed arrows indicate training‐only supervision. AP, arterial phase; DP, delayed phase; DWI, diffusion‐weighted imaging; H&E, hematoxylin and eosin; PVP, portal venous phase; VETC, vessels that encapsulate tumor clusters; WSI, whole‐slide image.

### 4.9 MRI‐Clinical Model Development

PIVOT‐Clinical was constructed as the final clinical risk model by combining the PIVOT‐MRI score with routinely available preoperative clinical variables. In the training cohort, the PIVOT‐MRI score was assessed together with AFP level, maximum tumor diameter, tumor number, cirrhosis, and viral hepatitis status. Univariable logistic regression was first used to examine associations with VETC status. Variables meeting the prespecified selection criterion were entered into multivariable logistic regression, and retained predictors were combined with the PIVOT‐MRI score to form PIVOT‐Clinical. The regression coefficients derived from the training cohort were fixed and applied without refitting to the internal test cohort and external validation cohorts.

### 4.10 Model Validation and Comparison

The performance of PIVOT‐MRI and PIVOT‐Clinical was evaluated in the internal test cohort and four external validation cohorts. Diagnostic performance was assessed using discrimination, calibration, and decision curve analysis. PIVOT‐Clinical was treated as the final clinical risk model, whereas PIVOT‐MRI was evaluated as the MRI‐derived component and the primary model for pathology‐informed alignment analysis.

To examine whether pathology‐informed embedding‐space alignment improved preoperative MRI assessment, PIVOT‐MRI was compared with MRI‐based deep learning models trained with the same MRI sequence set and the same CD34‐defined VETC label. These included ResNet3D‐MRI, Swin3D‐MRI, and Triad‐MRI [30, 31, 32]. Triad‐MRI used the same Triad‐SwinB initialization as PIVOT‐MRI but was trained without histopathologic embedding‐space alignment. All MRI‐based deep learning comparator models used the same training cohort, validation cohorts, and prespecified MRI preprocessing procedure as PIVOT‐MRI.

GigaPath‐H&E and GigaPath‐CD34 were prespecified as histopathologic comparator models. These models used Prov‐GigaPath‐derived slide‐level embeddings and the same patient‐level weakly supervised training strategy described above [26, 27]. Their performance was summarized in the Supporting Information because they used postoperative tissue images.

All comparison models were trained or specified in the training cohort and evaluated in the internal test cohort and external validation cohorts using the same prespecified analysis plan.

### 4.11 Ablation Study

Ablation analyses were performed to evaluate the contribution of histopathologic alignment, CD34‐derived vascular alignment, patient‐level MRI‐pathology pairing, and foundation‐model‐derived representations to MRI‐based VETC prediction. Each variant was trained in the training cohort and evaluated in the same internal and external validation cohorts as PIVOT‐MRI.

Starting from PIVOT‐MRI, variants were developed by omitting both H&E‐ and CD34‐derived alignment losses, omitting CD34‐derived vascular alignment, omitting H&E‐derived morphologic alignment, randomly mismatching paired MRI and pathology data, replacing GigaPath encoder‐derived pathology representations, and removing Triad initialization from the MRI encoder.

To examine whether the CD34‐derived vascular embedding provided patient‐specific vascular guidance rather than nonspecific regularization, supplementary control analyses compared the original model with shuffled CD34 embeddings and randomly mismatched MRI‐pathology pairs (Table S10).

To further assess the learned MRI‐pathology correspondence, cosine similarity was measured between paired MRI‐derived embeddings and the corresponding H&E‐derived morphologic or CD34‐derived vascular embeddings, with randomly mismatched MRI‐pathology pairs used as negative controls. Paired and mismatched similarities were compared in the internal test cohort.

### 4.12 Model Visualization and Interpretation

#### 4.12.1 SHAP‐Based Predictor Contribution

SHAP analysis was used to quantify the contribution of PIVOT‐MRI score and retained preoperative clinical variables in PIVOT‐Clinical. SHAP summary plots and individual explanation plots were generated in the training cohort and validation cohorts to assess the direction and relative magnitude of each predictor. This analysis was used to clarify whether PIVOT‐Clinical was primarily driven by the MRI‐derived PIVOT‐MRI score or by conventional clinical variables, without introducing manually assessed radiologic imaging features.

#### 4.12.2 MRI Activation Map Visualization

MRI activation maps were generated to visualize image regions contributing to VETC prediction [33]. Activation maps were produced from PIVOT‐MRI and overlaid on the corresponding MRI sequences. The maps were interpreted in relation to tumor margin, peritumoral parenchyma, and intratumoral enhancement heterogeneity. This analysis was performed to provide spatial interpretation of the MRI model while preserving the clinical‐use setting in which no pathology images are required for prediction.

#### 4.12.3 Biological Association and Pathway Enrichment

Exploratory biological association analyses were performed in an independent TCGA‐LIHC radiogenomic cohort. Following the strategy used in prior HCC radiopathomic studies that incorporated TCGA data for biological interpretation, TCGA‐LIHC cases were screened from The Cancer Genome Atlas and The Cancer Imaging Archive for paired pretreatment liver MRI and bulk RNA‐sequencing data. Patients were excluded if pretreatment MRI was unavailable or of insufficient quality, if the target HCC lesion could not be localized on MRI, or if RNA‐sequencing or clinical annotation was incomplete. Among potentially eligible TCGA‐LIHC imaging cases, 29 patients with both usable MRI and RNA‐sequencing data were included for exploratory biological analysis.

PIVOT‐MRI scores were generated from pretreatment MRI using the locked model and were not retrained using TCGA molecular or outcome data. Patients were stratified into high‐ and low‐PIVOT‐MRI‐score groups using the cutoff derived from the training cohort. This analysis was designed to examine whether the MRI‐derived VETC risk score was associated with vascular, proliferative, metabolic, and immune microenvironment programs, rather than to serve as an additional diagnostic validation cohort.

For transcriptomic analyses, differentially expressed genes between high‐ and low‐PIVOT‐MRI‐score groups were identified after normalization and multiple‐testing correction. Gene Ontology biological process and Kyoto Encyclopedia of Genes and Genomes pathway enrichment analyses were performed using ranked differentially expressed genes. Enrichment results were adjusted for multiple comparisons using the Benjamini‐Hochberg false discovery rate method. Immune and stromal abundance was estimated from bulk RNA‐sequencing profiles using MCP‐counter. Differences in inferred cell‐population abundance between PIVOT‐MRI‐defined risk groups were evaluated as exploratory biological correlates.

Biological association, immune infiltration, and pathway enrichment analyses in the TCGA cohort were considered exploratory because of the limited number of patients with paired MRI and RNA‐sequencing data.

### 4.13 Statistical Analysis

Continuous variables were reported as means and standard deviations or medians and interquartile ranges, and categorical variables were reported as numbers and percentages. Between‐group comparisons were performed using the Student t test, Mann‐Whitney U test, chi‐square test, or Fisher exact test, as appropriate. Univariable and multivariable logistic regression analyses were used to identify predictors of VETC status, with odds ratios, 95% CIs, and *p* values reported.

Statistical analysis and model reporting were planned with reference to TRIPOD+AI guidance for clinical prediction models [34]. Diagnostic performance was assessed using area under the receiver operating characteristic curve (AUC), accuracy, sensitivity, specificity, negative predictive value, and positive predictive value. 95% CIs for diagnostic metrics were calculated using bootstrap resampling. AUCs were compared using the paired DeLong test [35]. Calibration curves, Brier scores, and integrated calibration index were used to evaluate model calibration [36, 37]. Decision curve analysis was performed to estimate clinical net benefit across prespecified threshold probabilities [38]. The PIVOT‐Clinical cutoff was determined in the training cohort using the Youden index for VETC classification and was applied unchanged to stratify recurrence risk in all validation cohorts. Cohort‐specific curves were displayed over threshold probabilities of 0.10–0.60; the model‐positive clinical action and default strategy definitions are provided in Methods S8 and Figure S1.

Early recurrence‐free survival was estimated using the Kaplan‐Meier method and compared between score‐defined risk groups using the log‐rank test [39]. Unadjusted hazard ratios for high‐ vs. low‐score groups were estimated using Cox proportional hazards models. No multivariable adjustment was performed for the recurrence analyses. Two‐year recurrence rates were calculated for the high‐ and low‐score groups in the validation cohorts. A two‐sided *p* value less than 0.05 was considered to indicate statistical significance.

## Author Contributions

Z.S., W.L., N.J., and P.W. conceived and designed the study. W.L., W.Z., Y.L., X.L., J.Y., Y.Y., H.D., and W.C. contributed to patient enrollment, clinical data collection, imaging curation, histopathologic review, and follow‐up data acquisition. Z.S., W.L., S.W., and L.Q. contributed to model development, statistical analysis, and visualization. Z.S. and W.L. drafted the manuscript. N.J., W.C., and P.W. critically revised the manuscript. P.W. obtained funding and supervised the study. All authors reviewed and approved the final manuscript.

## Funding

This work was supported by the National Natural Science Foundation of China (No. 82227807), the Shanghai Municipal Science and Technology Commission (No. 25SF1908900), the Shanghai Municipal Commission of Health and Family Planning (No. 2022JC017), the Clinical Research Project of Tongji Hospital of Tongji University (No. ITJ(ZD)2301), and the Yunji Plan (No. KJ2‐0101‐25‐7262).

## Ethics Statement

This retrospective multicenter study was approved by the institutional review boards of all participating centers, including the Ethics Committee of Eastern Hepatobiliary Surgery Hospital, The Third Affiliated Hospital of Naval Medical University (EHBHKY2018‐1‐001), the Ethics Committee of Southwest Hospital, The First Affiliated Hospital of Army Medical University (KY2026114), and the Ethics Committee of Shuguang Hospital Affiliated to Shanghai University of Traditional Chinese Medicine (2023‐1249‐16‐01). The study was conducted in accordance with the Declaration of Helsinki. The requirement for written informed consent was waived because of the retrospective design and the use of deidentified data.

## Conflicts of Interest

The authors declare no conflicts of interest.

## Supporting information




**Supporting File**: advs78135‐sup‐0001‐SuppMat.docx.

## Data Availability

The data that support the findings of this study are not publicly available because of institutional restrictions, data‐use agreements, and patient privacy considerations. Deidentified data sufficient to reproduce selected aggregate analyses may be available from the corresponding author upon reasonable request, subject to approval by the participating institutions and completion of an appropriate data‐use agreement.
