# Supplementary material for: Foundation Model‐Enabled Pathology‐Informed MRI Model for Preoperative Prediction of Vessels Encapsulating Tumor Clusters in Resectable Hepatocellular Carcinoma: A Multicenter Retrospective Study
Source: Adv Sci (Weinh). 2026 Oct 18:e78135. Online ahead of print. doi: 10.1002/advs.78135 (PMC13676818; doi:10.1002/advs.78135)
Supplement: Supplementary file 1 [file ADVS-9999-e78135-s001.docx]

**Supporting Information**

**Foundation Model-Enabled Pathology-Informed MRI Model for Preoperative Prediction of Vessels Encapsulating Tumor Clusters in Resectable Hepatocellular Carcinoma: A Multicenter Retrospective Study**

Zhehan Shen^12†^, Wanmin Liu^12†^, Xiaoming Li^4†^, Wenjie Zou^3^, Yiman Li^4^, Jie Yuan^7^, Yasen Yimit^1,8^, Shihang Wang^9^, Linhao Qu^10^, Hui Dong^5^, Wei Chen^4*^, Ningyang Jia^6*^, and Peijun Wang^3*^

*Supplementary Methods • Supplementary Results • Supplementary Figure S1 • Supplementary Tables S1-S10*

# Supplementary Methods

## Supplementary Methods 1. Study population and data partitions

The primary unit of analysis for VETC classification was the patient. The study included 2052 patients: a development cohort from Eastern Hepatobiliary Surgery Hospital, The Third Affiliated Hospital of Naval Medical University, Yangpu District (EHBH-YP; n=869); an internal test cohort from EHBH-YP (n=374); and four external validation cohorts from Eastern Hepatobiliary Surgery Hospital, The Third Affiliated Hospital of Naval Medical University, Anting District (EHBH-AT; n=377); Southwest Hospital, The First Affiliated Hospital of Army Medical University (SWH; n=282); Tongji Hospital, School of Medicine, Tongji University (TJH; n=78), and Shuguang Hospital Affiliated to Shanghai University of Traditional Chinese Medicine (SGH; n=72). Cohort allocation, MRI–pathology linkage, model comparisons, and index-tumor localization analyses were conducted at the patient level, ensuring that data from the same patient were not assigned to different cohorts. Each patient in the validation cohorts contributed one index tumor to the localization analysis.

The development cohort was randomly partitioned at the patient level in an 8:2 ratio into a model-fitting subset (n=695) and a model-selection subset (n=174), with stratification by VETC status to preserve the class distribution across the two subsets. The model-fitting subset was used for parameter optimization, whereas the model-selection subset was used exclusively for checkpoint selection and early stopping. The internal test and external validation cohorts were reserved for final model evaluation and were not used for model fitting or model selection. The TCGA-LIHC radiotranscriptomic cohort included a separate set of 29 patients and was not used to fit or select PIVOT.

## Supplementary Methods 2. MRI inputs, preprocessing, and automated localization

The MRI input comprised T1-weighted imaging, fat-suppressed T2-weighted imaging, diffusion-weighted imaging, and arterial-, portal venous-, and delayed-phase contrast-enhanced images. Portal venous phase imaging served as the spatial reference. All six sequences were required. The lesion with the largest maximum diameter was designated as the index tumor in patients with multifocal HCC. Center- and sequence-specific acquisition parameters are summarized in Supplementary Table S1.

All sequences and the tumor mask were resampled to 1.5 × 1.5 × 3.0 mm. Non-reference sequences were rigidly registered to the portal venous phase image using mutual-information registration in SimpleITK. For each sequence, intensities were clipped at the 1st and 99th percentiles calculated within the tumor mask and then standardized using the mean and standard deviation of the clipped full volume. A fixed 96 × 160 × 160-voxel crop centered on the tumor-mask centroid was extracted and zero padded when it extended beyond the image boundary. The six processed volumes were stacked in a fixed sequence order.

Automated liver and index-tumor localization was evaluated separately using a three-dimensional full-resolution nnU-Net v2 model applied to portal venous phase images. Manual liver and index-tumor contours served as the reference standard, and tumor candidates were constrained to the liver region. Each validation patient contributed one index tumor. Successful identification of the manually annotated index tumor was evaluated at the patient level and stratified by maximum diameter (<20, 20-50, and >50 mm). Liver Dice similarity coefficient (DSC) was calculated in all validation patients, whereas index-tumor DSC was calculated among patients with successful automated localization.

## Supplementary Methods 3. Histopathologic processing and reference models

Histopathologic sampling followed a standardized seven-point sampling protocol, comprising one intratumoral sample, four tumor-margin samples containing both tumor and adjacent liver tissue, and two peritumoral samples obtained within 1 cm of the tumor. This routine sampling protocol was designed to capture spatial heterogeneity within and around the tumor. CD34 immunohistochemistry was not routinely performed on sections from all seven sampling sites. For VETC assessment, most patients had one tumor-containing CD34 whole-slide image, whereas a small minority had two; all available CD34-stained sections were derived from sampling sites containing tumor tissue.

Each patient contributed 4–10 H&E-stained histopathologic slides and, in most cases, one CD34 immunohistochemistry slide; only a small minority of patients had two CD34-stained slides. All available whole-slide images were digitized at 40× magnification. Slides with folding, tearing, severe blur, staining failure, or insufficient tumor-containing tissue were excluded. Whole-slide processing was performed without reference to VETC status and without manual selection of VETC-suspicious regions. Eligible tissue was divided into non-overlapping 256 × 256-pixel tiles, and tile coordinates were retained.

H&E and CD34 slides were processed by separate stain-specific reference models initialized from the same Prov-GigaPath weights. The pretrained tile and slide encoders generated a 768-dimensional representation for each slide. Representations from all eligible slides of one patient were combined by gated-attention pooling to obtain one patient-level embedding for each stain. The two reference models were trained separately using binary cross-entropy and AdamW for 50 epochs with a batch size of 16, a learning rate of 1 × 10^−4^, and weight decay of 1 × 10^−5^; the checkpoint with the highest validation AUC was retained. The reference encoders were then frozen during MRI model training.

## Supplementary Methods 4. PIVOT architecture, alignment, and optimization

Each of the six tumor-centered MRI volumes was encoded independently by a shared Triad-SwinB model initialized from the Triad-SwinB-SimMIM checkpoint. Multiscale Swin features were globally pooled, concatenated, and projected to a 768-dimensional sequence embedding. Sequence-specific residual adapters and learned sequence-type embeddings were applied before the six sequence tokens and a learned VETC token were passed through a two-layer, eight-head Transformer. The updated VETC token was used for classification and was mapped by two independent projection heads to the H&E and CD34 alignment spaces.

The classification term was unweighted binary cross-entropy. For each available patient-level histopathologic reference, the alignment error was defined as [max(1 − cosine similarity, 0)]³. The total objective was *L*_total_ = *L*_cls_ + 0.05*L*_morph_ + 0.10*L*_vasc_, with each alignment term averaged over patients with the corresponding stain.

Training was performed in two stages. During the first 20 epochs, the Triad encoder was frozen and the newly introduced MRI modules were optimized using the two alignment losses. During the subsequent 80 epochs, the classification loss was added and the final Swin stage was fine-tuned while earlier stages remained frozen. AdamW was used with a learning rate of 1 × 10^−4^ for newly introduced modules, 1 × 10^−5^ for the unfrozen backbone parameters, weight decay of 1 × 10^−5^, and a batch size of 2. The checkpoint with the highest validation AUC was retained. No stochastic MRI augmentation was applied. At inference, histopathologic images, reference encoders, and alignment heads were not required; prediction used the six preoperative MRI sequences only.

## Supplementary Methods 5. Clinical model, comparator models, and ablation analyses

Candidate variables for PIVOT-Clinical were the PIVOT-MRI score, alpha-fetoprotein, maximum tumor diameter, tumor number, cirrhosis, and viral hepatitis status. Univariable and multivariable logistic regression were performed in the development cohort. The final model combined the PIVOT-MRI score with maximum tumor diameter and alpha-fetoprotein, and its coefficients were fixed before evaluation in the internal and external test cohorts. The classification cutoff was selected in the development cohort by maximizing the Youden index and was applied unchanged in all validation analyses, including recurrence-risk stratification.

ResNet3D-MRI, Swin3D-MRI, and Triad-MRI used the same six MRI sequences, preprocessing, development data, VETC endpoint, and patient-level model-selection framework as PIVOT-MRI. Triad-MRI used the same Triad initialization without histopathologic alignment. Ablation variants changed one component at a time while retaining the remaining data and optimization pipeline. These analyses removed both alignment terms, removed either H&E or CD34 alignment, disrupted patient-level MRI–pathology pairing, evaluated dependence on Prov-GigaPath-derived pathology references, or removed Triad initialization. Paired and mismatched embedding correspondence was quantified with cosine similarity. Sensitivity analyses examined alternative morphologic and vascular alignment-loss weights.

## Supplementary Methods 6. Model interpretation and TCGA-LIHC analysis

SHAP values were used to summarize the direction and relative contribution of the PIVOT-MRI score and retained clinical variables in PIVOT-Clinical. Gradient-weighted class-activation maps from the locked PIVOT-MRI model were overlaid on the corresponding MRI sequences for spatial review of intratumoral, marginal, and peritumoral regions.

The exploratory TCGA-LIHC analysis included 29 patients with usable pretreatment liver MRI and matched bulk RNA-sequencing data. PIVOT-MRI scores were generated with the locked MRI model without molecular-data retraining, and the development-cohort cutoff was applied unchanged. Analyses included differential expression between high- and low-score groups, Spearman correlations with prespecified biological signatures, Gene Ontology biological-process and KEGG pathway enrichment, and MCP-counter estimates of immune and stromal abundance. Benjamini–Hochberg false-discovery-rate correction was applied separately within the differential-expression, signature-correlation, enrichment, and immune-cell testing families.

## Supplementary Methods 7. Statistical analysis

Unless otherwise specified, n denoted patients. Each patient contributed one index tumor to the localization analysis. Continuous variables were summarized as mean ± standard deviation or median (interquartile range), as appropriate, and categorical variables as counts and percentages. Two-group comparisons used the Student t test or Mann–Whitney U test for continuous variables and the χ² test or Fisher exact test for categorical variables.

Diagnostic performance was summarized by AUC, accuracy, sensitivity, specificity, positive predictive value, and negative predictive value. Ninety-five percent confidence intervals were calculated using 2000 patient-level bootstrap samples. AUCs for models evaluated in the same patients were compared using the paired DeLong test. Calibration was assessed using calibration curves, Brier score, and integrated calibration index. Decision-curve analysis compared model net benefit with treat-all and treat-none strategies over threshold probabilities of 0.10-0.60; the clinical action and strategy definitions are provided in Supplementary Methods 8.

Early recurrence-free survival was administratively censored at 24 months. The fixed PIVOT-Clinical cutoff selected for VETC classification defined the high- and low-score groups. Kaplan–Meier curves were compared using the two-sided log-rank test, and unadjusted Cox models with score group as the sole predictor were used to estimate hazard ratios and 95% confidence intervals. All tests were two-sided, with P<0.05 indicating statistical significance. Molecular analyses were interpreted at a false-discovery-rate threshold of q<0.05.

## Supplementary Methods 8. Clinical action and decision-curve interpretation

For decision-curve analysis, a model-positive result was defined as a predicted VETC probability at or above the threshold probability. The corresponding hypothetical clinical action was referral for multidisciplinary perioperative risk assessment, including review of tumor extent and liver reserve, together with planning of intensified postoperative imaging and alpha-fetoprotein surveillance after resection. This decision scenario was used to interpret net benefit and did not define surgical eligibility or selection of systemic treatment. Treat-all assigned every patient to this pathway, whereas treat-none assigned no patient.

Net benefit was calculated as TP/N - FP/N × pt/(1 - pt), where pt is the threshold probability. Cohort-specific curves were displayed from 0.10 to 0.60, spanning the decision range around the observed VETC prevalence in the validation cohorts. No single implementation threshold was selected from the decision curves.

# Supplementary Results

## Supplementary Results 1. Interobserver agreement for VETC classification.

Interobserver agreement for binary VETC classification (VETC-positive vs. VETC-negative) between the two pathologists before consensus was assessed separately at each participating center using Cohen’s κ coefficient. The analysis demonstrated high interobserver agreement, with Cohen’s κ values of 0.85 for EHBH-YP, 0.88 for EHBH-AT, 0.86 for SWH, 0.92 for TJH, and 0.93 for SGH.

## Supplementary Results 2. Automated MRI segmentation and index-tumor localization.

The predefined index tumor was successfully localized in 364 of 374 patients in the internal test cohort (97.3%; 95% CI, 95.1-98.5) and in 95.8%-97.5% of patients across the external validation cohorts. Liver DSC was evaluated in all validation patients, whereas index-tumor DSC was evaluated among successfully localized tumors. Detailed results by cohort and tumor size are provided in Supplementary Table S2.

## Supplementary Results 3. Expanded diagnostic performance.

Detailed diagnostic metrics, including AUC, accuracy, sensitivity, specificity, positive predictive value, and negative predictive value, are reported in Supplementary Table S3. Additional comparisons with histopathologic reference models are reported in Supplementary Table S6.

## Supplementary Results 4. Alignment validation and sensitivity analyses.

MRI-pathology correspondence was examined using paired-versus-mismatched embedding similarity, control analyses with disrupted pairing, and sensitivity analysis of alignment-loss weights. These results are summarized in Supplementary Tables S5, S9-10.

## Supplementary Results 5. Calibration, recurrence stratification, and exploratory biological associations.

Cohort-specific calibration and decision curves for PIVOT-MRI and PIVOT-Clinical are shown in Supplementary Figure S1, and quantitative calibration metrics are provided in Supplementary Table S4. Across the prespecified 0.10-0.60 threshold range, both models generally provided greater net benefit than the default strategies; PIVOT-Clinical exceeded PIVOT-MRI throughout the internal, EHBH-AT, and SWH cohorts and across most thresholds in TJH and SGH. Early recurrence stratification by PIVOT-Clinical score is summarized in Supplementary Table S7. Exploratory biological associations of PIVOT-MRI score in the TCGA-LIHC radiotranscriptomic cohort are summarized in Supplementary Table S8.

# Supplementary Figures

**Supplementary Figure S1. Cohort-Specific Calibration and Decision-Curve Analysis**

The top row shows calibration curves for the internal test, EHBH-AT, SWH, TJH, and SGH cohorts, respectively. The dashed diagonal denotes perfect calibration; observed VETC probabilities are plotted against mean predicted probabilities for PIVOT-MRI and PIVOT-Clinical. The inset statistics report case-level Brier score and LOWESS-based integrated calibration index. The bottom row shows decision curves in the corresponding cohorts across threshold probabilities of 0.10-0.60. Net benefit is shown for PIVOT-MRI, PIVOT-Clinical, treat-all, and treat-none strategies. A model-positive result indicates referral to the perioperative risk-assessment and intensified-surveillance pathway defined in Supplementary Methods 8. EHBH-AT, Eastern Hepatobiliary Surgery Hospital-Anting; SWH, Southwest Hospital; TJH, Tongji Hospital; SGH, Shuguang Hospital; VETC, vessels that encapsulate tumor clusters.

**Supplementary Tables**

**Supplementary Table S1. MRI Acquisition Parameters**

| **Center** | **Scanner** | **Sequence** | **TR/TE (ms)** | **FOV (mm)** | **Matrix** | **Thickness (mm)** | **B value (s/mm²)** |
| --- | --- | --- | --- | --- | --- | --- | --- |
| EHBH-YP | GE Optima MR360 (1.5T); GE SIGNA EXCITE (1.5T) | T2WI; T1WI; DWI; AP; PVP; DP | 6000/85; 180/2.0; 4000/75; 3.7/1.7; 3.7/1.7; 3.7/1.7 | 420 × 420; 420 × 420; 420 × 420; 420 × 420; 420 × 420; 420 × 420 | 512 × 512; 512 × 512; 256 × 256; 512 × 512; 512 × 512; 512 × 512 | 5-8; 5-8; 5-8; 3-5; 3-5; 3-5 | 0/50/600 |
| EHBH-AT | GE Discovery 750W (3.0T) | T2WI; T1WI; DWI; AP; PVP; DP | 6500/75; 3.3/1.5; 7000/55; 3.3/1.5; 3.3/1.5; 3.3/1.5 | 380 × 380; 360 × 360; 380 × 380; 360 × 360; 360 × 360; 360 × 360 | 320 × 320; 512 × 512; 128 × 128; 512 × 512; 512 × 512; 512 × 512 | 4-6; 4-6; 4-6; 3-5; 3-5; 3-5 | 0/50/600 |
| SWH | Siemens TrioTim (3.0T) | T2WI; T1WI; DWI; AP; PVP; DP | 7000/85; 3.42/1.25; 5500/81; 3.42/1.25; 3.42/1.25; 3.42/1.25 | 260 × 340; 275 × 400; 282 × 374; 275 × 400; 275 × 400; 275 × 400 | 244 × 320; 220 × 320; 136 × 180; 220 × 320; 220 × 320; 220 × 320 | 6.0; 2.5; 5.5; 2.5; 2.5; 2.5 | 0/50/400/800 |
| TJH | Philips Ingenia (3.0T); Siemens VERIO (3.0T) | T2WI; T1WI; DWI; AP; PVP; DP | 3300/85; 3.9/1.2; 7500/45; 3.9/1.3; 3.9/1.3; 3.9/1.3 | 400 × 400; 400 × 400; 400 × 400; 400 × 400; 400 × 400; 400 × 400 | 320 × 320; 256 × 256; 256 × 200; 256 × 256; 256 × 256; 256 × 256 | 3-6; 3-6; 4-6; 2-5; 2-5; 2-5 | 0/50/800 |
| SGH | Siemens MAGNETOM Skyra (3.0T) | T2WI; T1WI; DWI; AP; PVP; DP | 1800/87; 4.2/2.0; 8000/52; 3.8/1.2; 3.8/1.2; 3.8/1.2 | 380 × 380; 380 × 380; 380 × 380; 380 × 380; 380 × 380; 380 × 380 | 320 × 320; 260 × 320; 120 × 256; 464 × 576; 464 × 576; 384 × 512 | 5.0; 2.5; 5.0; 2.5; 2.5; 2.5 | 0/50/800 |

*Note. Sequence-dependent acquisition values are listed in the order T2WI; T1WI; DWI; AP; PVP; DP. B values apply to DWI only. AP, arterial phase; DP, delayed phase; DWI, diffusion-weighted imaging; FOV, field of view; PVP, portal venous phase; TE, echo time; TR, repetition time.*

**Supplementary Table S2. Patient-Level Index-Tumor Localization and Automated Liver and Tumor Segmentation Performance Across Validation Cohorts**

| **Metric** | **Internal test cohort (EHBH-YP; n=374)** | **EHBH-AT (n=377)** | **SWH (n=282)** | **TJH (n=78)** | **SGH (n=72)** |
| --- | --- | --- | --- | --- | --- |
| Liver segmentation DSC | 0.957 (0.954, 0.960) | 0.961 (0.958, 0.964) | 0.944 (0.940, 0.948) | 0.932 (0.925, 0.938) | 0.948 (0.941, 0.955) |
| Index-tumor segmentation DSC | 0.873 (0.866, 0.880) | 0.876 (0.869, 0.883) | 0.861 (0.854, 0.868) | 0.845 (0.830, 0.861) | 0.858 (0.842, 0.873) |
| Index-tumor maximum diameter |  |  |  |  |  |
| <20 mm | 91.7 (77/84) [83.8, 95.9] | 89.6 (60/67) [80.0, 94.8] | 89.5 (17/19) [68.6, 97.1] | 100.0 (5/5) [56.6, 100.0] | 75.0 (9/12) [46.8, 91.1] |
| 20-50 mm | 99.0 (206/208) [96.6, 99.7] | 97.8 (179/183) [94.5, 99.1] | 97.2 (141/145) [93.1, 98.9] | 94.6 (35/37) [82.3, 98.5] | 100.0 (34/34) [89.8, 100.0] |
| >50 mm | 98.8 (81/82) [93.4, 99.8] | 96.9 (123/127) [92.2, 98.8] | 99.2 (117/118) [95.4, 99.9] | 100.0 (36/36) [90.4, 100.0] | 100.0 (26/26) [87.1, 100.0] |
| Overall patient-level index localization | 97.3 (364/374) [95.1, 98.5] | 96.0 (362/377) [93.5, 97.6] | 97.5 (275/282) [95.0, 98.8] | 97.4 (76/78) [91.1, 99.3] | 95.8 (69/72) [88.5, 98.6] |

*Note. DSC values are means (95% percentile-bootstrap CIs). Each validation patient contributed one index tumor, defined as the tumor with the largest maximum diameter according to the manual reference contour. Patient-level index-tumor localization is reported as percentage (successfully localized index tumors/all index tumors) [95% Wilson score CI]. Size strata were based on index-tumor maximum diameter; values of 20 and 50 mm were included in the 20-50-mm stratum. Liver DSC was calculated in all validation patients. Index-tumor DSC was calculated only in successfully localized index tumors (internal test, n=364; EHBH-AT, n=362; SWH, n=275; TJH, n=76; SGH, n=69). DSC, Dice similarity coefficient; CI, confidence interval.*

**Supplementary Table S3. Detailed Diagnostic Performance of MRI-Based Deep Learning Models, the Clinical-Only Model, PIVOT-MRI, and PIVOT-Clinical**

| **Model** | **Cohort** | **AUC** | **Accuracy** | **Sensitivity** | **Specificity** | **NPV** | **PPV** |
| --- | --- | --- | --- | --- | --- | --- | --- |
| ResNet3D-MRI | EHBH-YP internal test | 0.813 (0.764, 0.862) | 0.735 (275/374) [0.688, 0.777] | 0.758 (97/128) [0.677, 0.824] | 0.724 (178/246) [0.665, 0.776] | 0.852 (178/209) [0.797, 0.893] | 0.588 (97/165) [0.512, 0.660] |
| ResNet3D-MRI | EHBH-AT | 0.779 (0.727, 0.831) | 0.748 (282/377) [0.702, 0.789] | 0.748 (86/115) [0.661, 0.818] | 0.748 (196/262) [0.692, 0.797] | 0.871 (196/225) [0.821, 0.909] | 0.566 (86/152) [0.486, 0.642] |
| ResNet3D-MRI | SWH | 0.789 (0.729, 0.849) | 0.755 (213/282) [0.702, 0.802] | 0.747 (68/91) [0.649, 0.825] | 0.759 (145/191) [0.694, 0.814] | 0.863 (145/168) [0.803, 0.907] | 0.596 (68/114) [0.505, 0.682] |
| ResNet3D-MRI | TJH | 0.758 (0.648, 0.868) | 0.756 (59/78) [0.651, 0.838] | 0.724 (21/29) [0.543, 0.853] | 0.776 (38/49) [0.641, 0.870] | 0.826 (38/46) [0.693, 0.909] | 0.656 (21/32) [0.483, 0.796] |
| ResNet3D-MRI | SGH | 0.766 (0.656, 0.876) | 0.750 (54/72) [0.639, 0.836] | 0.731 (19/26) [0.539, 0.863] | 0.761 (35/46) [0.621, 0.861] | 0.833 (35/42) [0.694, 0.917] | 0.633 (19/30) [0.455, 0.781] |
| Swin3D-MRI | EHBH-YP internal test | 0.842 (0.796, 0.888) | 0.778 (291/374) [0.733, 0.817] | 0.797 (102/128) [0.719, 0.857] | 0.768 (189/246) [0.712, 0.817] | 0.879 (189/215) [0.829, 0.916] | 0.642 (102/159) [0.564, 0.712] |
| Swin3D-MRI | EHBH-AT | 0.816 (0.767, 0.865) | 0.793 (299/377) [0.749, 0.831] | 0.809 (93/115) [0.727, 0.870] | 0.786 (206/262) [0.733, 0.832] | 0.904 (206/228) [0.858, 0.935] | 0.624 (93/149) [0.544, 0.698] |
| Swin3D-MRI | SWH | 0.806 (0.748, 0.864) | 0.794 (224/282) [0.743, 0.837] | 0.791 (72/91) [0.697, 0.862] | 0.796 (152/191) [0.733, 0.847] | 0.889 (152/171) [0.833, 0.928] | 0.649 (72/111) [0.556, 0.731] |
| Swin3D-MRI | TJH | 0.785 (0.675, 0.895) | 0.782 (61/78) [0.678, 0.859] | 0.759 (22/29) [0.579, 0.878] | 0.796 (39/49) [0.664, 0.885] | 0.848 (39/46) [0.718, 0.924] | 0.688 (22/32) [0.514, 0.820] |
| Swin3D-MRI | SGH | 0.796 (0.686, 0.906) | 0.792 (57/72) [0.684, 0.869] | 0.769 (20/26) [0.579, 0.890] | 0.804 (37/46) [0.668, 0.893] | 0.860 (37/43) [0.727, 0.934] | 0.690 (20/29) [0.508, 0.827] |
| Triad-MRI | EHBH-YP internal test | 0.835 (0.788, 0.882) | 0.767 (287/374) [0.722, 0.807] | 0.797 (102/128) [0.719, 0.857] | 0.752 (185/246) [0.694, 0.802] | 0.877 (185/211) [0.826, 0.915] | 0.626 (102/163) [0.549, 0.696] |
| Triad-MRI | EHBH-AT | 0.812 (0.763, 0.861) | 0.790 (298/377) [0.747, 0.829] | 0.791 (91/115) [0.708, 0.856] | 0.790 (207/262) [0.737, 0.835] | 0.896 (207/231) [0.850, 0.929] | 0.623 (91/146) [0.542, 0.698] |
| Triad-MRI | SWH | 0.803 (0.745, 0.861) | 0.787 (222/282) [0.736, 0.831] | 0.780 (71/91) [0.685, 0.853] | 0.791 (151/191) [0.727, 0.842] | 0.883 (151/171) [0.826, 0.923] | 0.640 (71/111) [0.547, 0.723] |
| Triad-MRI | TJH | 0.781 (0.671, 0.891) | 0.769 (60/78) [0.664, 0.849] | 0.759 (22/29) [0.579, 0.878] | 0.776 (38/49) [0.641, 0.870] | 0.844 (38/45) [0.712, 0.923] | 0.667 (22/33) [0.496, 0.802] |
| Triad-MRI | SGH | 0.795 (0.685, 0.905) | 0.778 (56/72) [0.669, 0.858] | 0.769 (20/26) [0.579, 0.890] | 0.783 (36/46) [0.644, 0.877] | 0.857 (36/42) [0.722, 0.933] | 0.667 (20/30) [0.488, 0.808] |
| Clinical-only | EHBH-YP internal test | 0.670 (0.611, 0.725) | 0.559 (209/374) [0.508, 0.608] | 0.758 (97/128) [0.677, 0.824] | 0.455 (112/246) [0.394, 0.518] | 0.783 (112/143) [0.709, 0.843] | 0.420 (97/231) [0.358, 0.484] |
| Clinical-only | EHBH-AT | 0.649 (0.587, 0.707) | 0.517 (195/377) [0.467, 0.567] | 0.791 (91/115) [0.708, 0.856] | 0.397 (104/262) [0.340, 0.457] | 0.812 (104/128) [0.736, 0.871] | 0.365 (91/249) [0.308, 0.427] |
| Clinical-only | SWH | 0.651 (0.578, 0.720) | 0.465 (131/282) [0.407, 0.523] | 0.824 (75/91) [0.733, 0.889] | 0.293 (56/191) [0.233, 0.361] | 0.778 (56/72) [0.669, 0.858] | 0.357 (75/210) [0.295, 0.424] |
| Clinical-only | TJH | 0.621 (0.486, 0.749) | 0.526 (41/78) [0.416, 0.633] | 0.759 (22/29) [0.579, 0.878] | 0.388 (19/49) [0.264, 0.528] | 0.731 (19/26) [0.539, 0.863] | 0.423 (22/52) [0.299, 0.558] |
| Clinical-only | SGH | 0.631 (0.492, 0.767) | 0.556 (40/72) [0.441, 0.665] | 0.808 (21/26) [0.621, 0.915] | 0.413 (19/46) [0.283, 0.557] | 0.792 (19/24) [0.595, 0.908] | 0.438 (21/48) [0.307, 0.577] |
| PIVOT-MRI | EHBH-YP internal test | 0.887 (0.847, 0.926) | 0.821 (307/374) [0.779, 0.856] | 0.828 (106/128) [0.753, 0.884] | 0.817 (201/246) [0.764, 0.860] | 0.901 (201/223) [0.855, 0.934] | 0.702 (106/151) [0.625, 0.769] |
| PIVOT-MRI | EHBH-AT | 0.861 (0.816, 0.906) | 0.841 (317/377) [0.801, 0.874] | 0.826 (95/115) [0.747, 0.885] | 0.847 (222/262) [0.799, 0.886] | 0.917 (222/242) [0.876, 0.946] | 0.704 (95/135) [0.622, 0.774] |
| PIVOT-MRI | SWH | 0.858 (0.806, 0.910) | 0.844 (238/282) [0.797, 0.882] | 0.824 (75/91) [0.733, 0.889] | 0.853 (163/191) [0.796, 0.897] | 0.911 (163/179) [0.860, 0.944] | 0.728 (75/103) [0.635, 0.805] |
| PIVOT-MRI | TJH | 0.839 (0.736, 0.942) | 0.821 (64/78) [0.721, 0.890] | 0.793 (23/29) [0.616, 0.902] | 0.837 (41/49) [0.710, 0.915] | 0.872 (41/47) [0.748, 0.940] | 0.742 (23/31) [0.568, 0.863] |
| PIVOT-MRI | SGH | 0.852 (0.749, 0.955) | 0.833 (60/72) [0.731, 0.902] | 0.808 (21/26) [0.621, 0.915] | 0.848 (39/46) [0.718, 0.924] | 0.886 (39/44) [0.760, 0.950] | 0.750 (21/28) [0.566, 0.873] |
| PIVOT-Clinical | EHBH-YP internal test | 0.904 (0.867, 0.941) | 0.853 (319/374) [0.813, 0.885] | 0.844 (108/128) [0.771, 0.897] | 0.858 (211/246) [0.809, 0.896] | 0.913 (211/231) [0.870, 0.943] | 0.755 (108/143) [0.679, 0.818] |
| PIVOT-Clinical | EHBH-AT | 0.884 (0.844, 0.924) | 0.822 (310/377) [0.780, 0.858] | 0.800 (92/115) [0.718, 0.863] | 0.832 (218/262) [0.782, 0.872] | 0.905 (218/241) [0.861, 0.936] | 0.676 (92/136) [0.594, 0.749] |
| PIVOT-Clinical | SWH | 0.873 (0.824, 0.922) | 0.819 (231/282) [0.770, 0.860] | 0.802 (73/91) [0.709, 0.871] | 0.827 (158/191) [0.767, 0.874] | 0.898 (158/176) [0.844, 0.934] | 0.689 (73/106) [0.595, 0.769] |
| PIVOT-Clinical | TJH | 0.851 (0.752, 0.950) | 0.808 (63/78) [0.707, 0.880] | 0.793 (23/29) [0.616, 0.902] | 0.816 (40/49) [0.686, 0.900] | 0.870 (40/46) [0.743, 0.939] | 0.719 (23/32) [0.546, 0.844] |
| PIVOT-Clinical | SGH | 0.861 (0.762, 0.962) | 0.792 (57/72) [0.684, 0.869] | 0.769 (20/26) [0.579, 0.890] | 0.804 (37/46) [0.668, 0.893] | 0.860 (37/43) [0.727, 0.934] | 0.690 (20/29) [0.508, 0.827] |

*Note.—AUC values are shown with 95% percentile-bootstrap CIs. Accuracy, sensitivity, specificity, NPV, and PPV are shown as proportions (numerator/denominator) [95% Wilson score CI]. AUC, area under the receiver operating characteristic curve; CI, confidence interval; NPV, negative predictive value; PPV, positive predictive value.*

**Supplementary Table S4. Calibration Metrics for PIVOT-MRI and PIVOT-Clinical**

| **Model** | **Cohort** | **Brier score** | **ICI** | **Calibration intercept (95% CI)** | **Calibration slope (95% CI)** |
| --- | --- | --- | --- | --- | --- |
| PIVOT-MRI | EHBH-YP internal test | 0.139 | 0.093 | -0.57 (-0.89, -0.30) | 1.56 (1.28, 1.93) |
| PIVOT-MRI | EHBH-AT | 0.151 | 0.110 | -0.63 (-0.92, -0.36) | 1.54 (1.21, 1.97) |
| PIVOT-MRI | SWH | 0.158 | 0.130 | -0.76 (-1.17, -0.44) | 1.59 (1.26, 2.08) |
| PIVOT-MRI | TJH | 0.168 | 0.089 | -0.38 (-0.94, 0.18) | 1.46 (0.76, 2.64) |
| PIVOT-MRI | SGH | 0.164 | 0.120 | -0.65 (-1.47, -0.03) | 1.77 (1.11, 2.95) |
| PIVOT-Clinical | EHBH-YP internal test | 0.126 | 0.065 | -0.42 (-0.74, -0.14) | 1.43 (1.16, 1.80) |
| PIVOT-Clinical | EHBH-AT | 0.137 | 0.100 | -0.56 (-0.84, -0.28) | 1.54 (1.19, 2.00) |
| PIVOT-Clinical | SWH | 0.144 | 0.112 | -0.64 (-0.99, -0.32) | 1.56 (1.24, 1.99) |
| PIVOT-Clinical | TJH | 0.154 | 0.072 | -0.34 (-0.97, 0.23) | 1.33 (0.80, 2.29) |
| PIVOT-Clinical | SGH | 0.150 | 0.062 | -0.17 (-0.83, 0.41) | 1.54 (1.03, 2.55) |

*Note. Calibration intercept and slope were estimated by logistic recalibration of the logit of predicted probability. Ideal values are 0 for the intercept and 1 for the slope. ICI was the mean absolute difference between predicted probability and a LOWESS calibration estimate (span 2/3, no robust iterations). Confidence intervals were obtained from 1,000 patient-level bootstrap resamples within each cohort. CI, confidence interval; ICI, integrated calibration index.*

**Supplementary Table S5. Cosine Similarity Between MRI and Histopathologic Features**

| **Feature comparison** | **Cohort** | **Paired MRI-pathology features** | **Randomly mismatched features** | **P value** |
| --- | --- | --- | --- | --- |
| MRI-H&E morphologic | EHBH-YP internal test | 0.67 (0.64, 0.70) | 0.32 (0.29, 0.35) | <0.001 |
| MRI-CD34 vascular | EHBH-YP internal test | 0.71 (0.68, 0.74) | 0.34 (0.31, 0.37) | <0.001 |

**Supplementary Table S6. Diagnostic Performance of PIVOT-MRI, GigaPath-H&E, and GigaPath-CD34**

| **Model** | **EHBH-YP internal test AUC** | **EHBH-AT AUC** | **SWH AUC** | **TJH AUC** | **SGH AUC** | **P value*** |
| --- | --- | --- | --- | --- | --- | --- |
| PIVOT-MRI | 0.887 (0.847, 0.926) | 0.861 (0.816, 0.906) | 0.858 (0.806, 0.910) | 0.839 (0.736, 0.942) | 0.852 (0.749, 0.955) | / |
| GigaPath-H&E | 0.916 (0.881, 0.951) | 0.895 (0.858, 0.932) | 0.886 (0.840, 0.932) | 0.871 (0.778, 0.966) | 0.879 (0.782, 0.976) | <0.001 |
| GigaPath-CD34 | 0.973 (0.955, 0.991) | 0.961 (0.939, 0.983) | 0.955 (0.928, 0.982) | 0.947 (0.899, 0.995) | 0.951 (0.899, 1.000) | <0.001 |

*Note. *P values compare AUCs between each histopathologic model and PIVOT-MRI in the internal test cohort using the paired DeLong test.*

**Supplementary Table S7. Early Recurrence Stratification by PIVOT-Clinical Score**

| **Cohort set** | **HR (95% CI)** | **High PIVOT-Clinical score 2-year recurrence rate** | **Low PIVOT-Clinical score 2-year recurrence rate** | **Log-rank P value** |
| --- | --- | --- | --- | --- |
| EHBH-YP internal test | 2.43 (1.60, 3.70) | 38.4% | 18.4% | <0.001 |
| EHBH-AT | 2.40 (1.58, 3.65) | 40.0% | 21.5% | <0.001 |
| SWH | 2.97 (1.93, 4.56) | 55.1% | 25.3% | <0.001 |
| TJH | 2.67 (1.13, 6.33) | 44.9% | 22.2% | 0.025 |
| SGH | 2.60 (1.06, 6.36) | 44.0% | 22.7% | 0.037 |

*Note. Two-year recurrence rates are 1 minus the Kaplan-Meier estimate at 24 months. The fixed development-cohort VETC classification cutoff was applied unchanged in all validation cohorts. CI, confidence interval; HR, hazard ratio.*

**Supplementary Table S8. Exploratory Biological Associations of PIVOT-MRI Score**

| **Analysis domain** | **Association with PIVOT-MRI score** | **Effect summary** | **Adjusted P value** |
| --- | --- | --- | --- |
| Differential expression | Genes differentially expressed between high- and low-score groups | 496 genes after FDR correction | FDR <0.05 |
| Signature correlation | Hypoxia-angiogenesis signature | Spearman r, 0.621 | 0.001 |
| Signature correlation | Vascular endothelium signature | Spearman r, 0.482 | 0.015 |
| Signature correlation | Proliferation signature | Spearman r, 0.573 | 0.003 |
| Signature correlation | Immune-checkpoint macrophage signature | Spearman r, 0.420 | 0.032 |
| Signature correlation | Hepatic metabolism signature | Spearman r, -0.730 | <0.001 |
| Signature correlation | Cytochrome P450/xenobiotic metabolism signature | Spearman r, -0.651 | <0.001 |
| Immune microenvironment | Higher monocytic-lineage abundance in high-score group by MCP-counter | Median score, 3.40 vs 2.59; P=0.007 | 0.071 |
| GO biological process | Regulation of cell-cycle phase transition | NES, 1.62 | <0.001 |
| GO biological process | TGF-beta response | NES, 1.31 | 0.023 |
| GO biological process | Extracellular matrix organization | NES, 1.26 | 0.057 |
| KEGG pathway | Cell cycle | NES, 1.76 | <0.001 |
| KEGG pathway | Proteoglycans in cancer | NES, 1.48 | <0.001 |
| KEGG pathway | Focal adhesion | NES, 1.43 | 0.002 |
| KEGG pathway | PI3K-Akt signaling pathway | NES, 1.24 | 0.022 |
| KEGG pathway | Fatty acid degradation, enriched in low-score group | NES, -3.90 | <0.001 |
| KEGG pathway | Primary bile acid biosynthesis, enriched in low-score group | NES, -3.31 | <0.001 |
| KEGG pathway | PPAR signaling pathway, enriched in low-score group | NES, -3.09 | <0.001 |
| KEGG pathway | Drug metabolism-cytochrome P450, enriched in low-score group | NES, -2.97 | <0.001 |

**Supplementary Table S9. Sensitivity Analysis of Alignment-Loss Weights**

| ***λ*_morph_** | ***λ*_vasc_** | **Interpretation** | **Internal test AUC (95% CI)** | **Mean external AUC** |
| --- | --- | --- | --- | --- |
| 0 | 0 | No histopathologic alignment | 0.835 (0.788, 0.882) | 0.797 |
| 0.05 | 0 | H&E morphologic alignment only | 0.858 (0.814, 0.902) | 0.819 |
| 0 | 0.10 | CD34 vascular alignment only | 0.879 (0.838, 0.920) | 0.837 |
| 0.05 | 0.05 | Balanced low-weight dual alignment | 0.882 (0.842, 0.922) | 0.839 |
| 0.05 | 0.10 | PIVOT-MRI setting | 0.887 (0.847, 0.926) | 0.853 |
| 0.10 | 0.10 | Balanced moderate-weight dual alignment | 0.883 (0.843, 0.923) | 0.840 |
| 0.10 | 0.05 | Morphology-dominant alignment | 0.875 (0.834, 0.916) | 0.832 |
| 0.05 | 0.15 | Stronger vascular-dominant alignment | 0.884 (0.845, 0.923) | 0.841 |
| 0.25 | 0.25 | High-weight dual alignment | 0.867 (0.824, 0.910) | 0.823 |

**Supplementary Table S10. Control Analyses for MRI-Pathology Alignment**

| **Analysis** | **Cohort set** | **Result** | **Interpretation** |
| --- | --- | --- | --- |
| Shuffled CD34 embedding | Internal and external validation cohorts | Mean external AUC, 0.821; delta vs PIVOT, -0.032; P=0.004 | Correct patient-level CD34 correspondence was required for the vascular-alignment gain |
| Mismatched MRI-pathology pairs | Internal and external validation cohorts | Mean external AUC, 0.810; delta vs PIVOT, -0.043; P<0.001 | Alignment benefit was not explained by nonspecific regularization from pathology embeddings |
